# Mechanistic Insights Into Sustainable Catalytic Pyrolysis of Biomass‐Derived Ferulic Acid: The Role of Alumina Regeneration

**DOI:** 10.1002/chem.71397

**Published:** 2026-07-14

**Authors:** Tetiana V. Kulik, Borys B. Palianytsia, Mykola M. Ilchenko, Nataliia S. Nastasiienko, David J. Morgan, Liudmila P. Storozhuk, David J. Willock, Greg Shaw, Duncan F. Wass, Rafael Luque, Philip R. Davies, Mykola T. Kartel

**Affiliations:** ^1^ Cardiff Catalysis Institute School of Chemistry Translational Research Hub Cardiff University Cardiff Wales UK; ^2^ Chuiko Institute of Surface Chemistry of National Academy of Science of Ukraine Kyiv Ukraine; ^3^ Institute of Molecular Biology and Genetics National Academy of Science of Ukraine Kyiv Ukraine; ^4^ Biophysics Group Department of Physics and Astronomy University College London London UK; ^5^ UCL Healthcare Biomagnetics and Nanomaterials Laboratories London UK; ^6^ National University of Science and Technology Polytehnica Bucharest Bucharest Romania; ^7^ Universidad ECOTEC Samborondón Ecuador

**Keywords:** 4‐vinylguaiacol, biomass conversion, kinetics and mechanisms, lignin, pyrolysis oil

## Abstract

A fundamental understanding of catalyst deactivation and regeneration is essential for developing sustainable catalytic pyrolysis processes for lignocellulosic biomass conversion. Herein, the effect of alumina regeneration on the catalytic pyrolysis of ferulic acid, a lignin‐derived model compound, was investigated using kinetic analysis combined with FTIR, TPD–MS, XPS, TEM, XRD, thermogravimetry, and DFT calculations. Comparative kinetic analysis showed that the apparent activation energies of key reactions, including decarboxylation, demethoxylation, and the formation of 4‐vinylguaiacol, guaiacol, phenol, and other aromatic products, increase by 4–23 kJ mol^−^^1^ after catalyst regeneration, indicating partial modification of the alumina surface. DFT calculations revealed that 4‐vinylguaiacol formation proceeds preferentially via a surface‐assisted acidic decarboxylation pathway involving alumina‐bound intermediates, whereas alternative surface interactions generate strongly bound species that may contribute to catalyst deactivation. Combined experimental and theoretical evidence indicates that surface‐bound phenolate complexes act as precursors to carbon deposition. These species undergo styrene‐like polymerization on alumina, forming a polymeric carbonaceous layer that decomposes upon heating, releasing aromatic products and progressively forming extended polyaromatic carbon domains. The proposed mechanism is supported by TEM, FTIR, XPS, and TPD–MS results. These findings establish a unified mechanistic framework linking catalytic decarboxylation, carbon‐layer formation, and catalyst deactivation during biomass conversion.

## Graphical abstract

Surface‐bound ferulate phenolate complexes undergo styrene‐like polymerization on alumina, yielding a polymeric carbonaceous layer that progressively transforms into polyaromatic carbon domains with concomitant release of aromatic products, while parallel surface‐assisted decarboxylation produces 4‐vinylguaiacol.

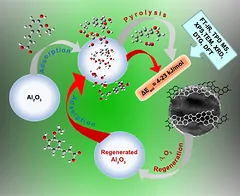

## 1 Introduction

The catalytic conversion of second‐generation non‐food biomass (2G biomass) and agricultural waste into sustainable platform chemicals and transportation biofuels through pyrolysis has been widely explored over the past few decades [1, 2, 3, 4, 5, 6, 7, 8, 9, 10]. The transition toward sustainable development and the “decarbonization” of the economy necessitate significant advancements and the accelerated implementation of efficient and economically viable biomass processing technologies [5, 6, 7, 8, 9, 10] such as catalytic pyrolysis [1, 2, 3, 4, 7, 11].

Catalytic pyrolysis technologies aim to develop highly efficient catalysts that can withstand multiple cycles of the process without losing activity and, importantly, can be easily regenerated. Catalyst replacement and process shutdown cost the industry billions of dollars annually [12]. As a result, studies focused on understanding the nature of catalyst deactivation mechanisms in specific biomass 2G pyrolysis processes hold significant practical value [11]. A deeper understanding of catalyst poisoning and deactivation mechanisms at the molecular level will facilitate the development of more durable catalysts with extended lifespans, while also enabling the creation of effective catalyst regeneration methods. Catalyst deactivation remains a critical area of research, both academically and industrially.

Several key catalyst deactivation mechanisms have been recently reviewed and described in detail [12]. In that review, catalyst deactivation was systematically classified by type (chemical, thermal, and mechanical) and by mechanism (poisoning, contamination, thermal decomposition, gas‐phase reactions, reactions at the gas‐solid interface, solid‐phase reactions, and mechanical degradation such as abrasion or crushing) [12]. Additionally, common catalytic poisons were categorized based on their chemical structure [12]. In the context of biomass pyrolysis, the most critical deactivation agents are likely to be compounds containing double and triple carbon–carbon or carbon–oxygen bonds, such as acetylenes, ketenes, alkenes, hydrogen cyanide (HCN), aromatics, and other unsaturated hydrocarbons, which are invariably present in pyrolysis vapors from biomass, its components, and model compounds [13, 14, 15, 16, 17]. Among these, the formation of carbonaceous deposits (or coke) on the catalyst surface is particularly detrimental, as it blocks the active sites and significantly reduces catalytic activity [18]. This issue is exacerbated by the oxygen‐free, vacuum conditions under which pyrolysis typically occurs. The analysis of these carbon deposits requires not only conventional analytical techniques but also advanced methods, complemented by theoretical calculations [18, 19, 20]. Moreover, this aspect is particularly important, as such carbon coatings, including graphene‐like layers, can serve as catalytically active supports, Verma et al. [20].

Second‐generation biomass is considered one of the most promising, large‐scale, and renewable resources for green conversion into valuable chemicals, renewable biofuels, and biochar via catalytic pyrolysis. This type of biomass is a complex, multicomponent system that can be broadly divided into three main groups of components, interconnected through various covalent and supramolecular bonds: [9, 10, 17] (1) carbohydrate components (mono‐, oligo‐, and polysaccharides); (2) extractives (triglycerides, fatty acids, etc.); and (3) lignin‐derived components (lignins, lignols, and cinnamic acid derivatives) [13, 14, 15, 17]. The catalytic pyrolysis of these components is inherently complex due to several factors: (1) each component transforms over different temperature ranges; (2) they yield diverse product distributions; (3) different catalyst types are required for optimal conversion of each component; (4) catalyst deactivation (poisoning) occurs through distinct mechanisms, depending on both the catalyst material and the chemical nature of the pyrolysis reactions. This complexity underscores the need for a tailored catalytic approach and a profound understanding of the chemical behavior of individual biomass fractions to facilitate efficient and selective pyrolytic conversion.

A promising approach to elucidating the mechanisms of second‐generation (2G) biomass pyrolysis and catalyst deactivation during catalytic pyrolysis involves the use of biomass model compounds, [13, 14, 15, 17] particularly lignin‐derived model compounds such as ferulic acid [21], since lignin is among the most challenging biomass components to process [9, 10]. Given that lignin constitutes 10%–30% of lignocellulosic biomass and its accumulation as waste poses significant environmental concerns [10, 22, 23, 24], the scale of its presence is notable—particularly as the paper industry alone generates approximately 70 million tons of lignin waste annually [24]. In kraft pulp mills, lignin is not merely a waste byproduct but represents an essential renewable carbon resource used in recovery boilers for the regeneration of cooking chemicals and for heat and steam production. Nevertheless, a significant fraction of lignin (typically up to ∼20%) can be separated from black liquor and further valorized into high‐value chemicals and functional materials without compromising mill operation. Efficient processing of this low‐cost, lignin‐rich 2G biomass can lead to the production of bio‐oil and high‐value chemical products [25, 26, 27]. Moreover, substantial amounts of ferulic acid can be sustainably recovered from agro‐industrial residues, particularly cereal bran, using green extraction and biorefinery approaches. Global annual production of wheat and rye bran is estimated at approximately 74.8–112.1 Mt, and continues to increase, highlighting a readily available and renewable feedstock. Recent advances in pretreatment strategies, including enzymatic hydrolysis and downstream processing, have enabled ferulic acid yields of up to 8.6–11.3 g kg^−^^1^ from such biomass residues [28].

The possibility of regenerating nanosized alumina after the pyrolysis of ferulic acid, a lignin model compound that simulates the biomass of coniferous tree species, was explored in this study. The processes of catalyst deactivation and regeneration were investigated using XPS, XRD, TEM, FTIR, thermogravimetry, and DFT. Kinetic studies were conducted to compare the catalytic activity of the initial [29] and regenerated catalysts, with kinetic parameters being obtained through temperature‐programmed desorption mass spectrometry (TPD‐MS) [30, 31, 32, 33, 34, 35]. In addition, we carried out a DFT investigation of the decarboxylation of ferulic acid leading to the formation of the value‐added product 4‐vinyl guaiacol, accompanied by an analysis and comparison of the experimental activation energies for this transformation on the surfaces of both fresh and regenerated catalysts. Notably, 4‐vinylguaiacol and other bio‐based vinyl phenols serve as versatile monomeric building blocks [36, 37, 38] for the synthesis of actual pharmaceutical products, natural compounds with pharmacological activity, drug‐leads, new bio‐based polymers, and other high‐value materials that can replace products derived from nonrenewable resources. In addition, 4‐vinyl guaiacol is widely used in the food industry as a flavoring compound that enhances sensory quality by imparting characteristic clove‐like, spicy, and smoky notes [38, 39]. It is a naturally occurring aroma constituent in a broad range of food and beverage products, including baked goods, soy sauces, meat products, wine, whisky, and beer, etc., where it often plays a key role in defining the desired flavor profile. Owing to its functional versatility and demand, 4‐vinylguaiacol is a high‐value product; for example, Sigma–Aldrich (Merck) lists it at approximately USD 2,080 kg^−^^1^ (USD 223 per 25 g) for 98% food‐grade quality, underscoring the economic attractiveness of its production from biomass‐derived ferulic acid.

4‐Vinylguaiacol and other bio‐based styrenes can be produced via thermochemical or biochemical catalytic routes from lignin‐containing feedstocks, lignin‐derived ferulic acid and other related *p*‐hydroxycinnamic acids [36]. Consequently, substantial research efforts are focused on optimizing production conditions and developing efficient technological pathways for their manufacture [40, 41, 42, 43]. The feasibility of obtaining these compounds through direct and catalytic pyrolysis of both *p*‐hydroxycinnamic acids and real lignocellulosic biomass has been demonstrated in our earlier studies [14, 15, 16, 17].

## 2 Results and Discussion

The absence of oxygen and the vacuum conditions during pyrolysis lead to the rapid formation of various carbon coatings on the catalyst surface, including amorphous carbon, condensed polyaromatic structures, and graphite‐like coatings. Furthermore, the regeneration process may not always fully restore catalyst activity due to the formation of firmly bound species, such as surface carbides and carbonates, that block the active sites. High temperatures used during regeneration can also negatively impact catalytic performance, as surface dehydration may occur, reducing the proportion of catalytically active surface groups. After pyrolysis of ferulic acid (Fer) over alumina, the sample turned black, indicating the formation of a carbon coating on the surface. To regenerate the catalyst, the sample was calcined for 2 h in air at 500°C, which successfully removed the carbon coating, restoring its appearance to a snow‐white color (Figure S1, Supporting Information). Following this, various methods were employed to investigate the nature of the carbon coating on the catalyst surface. Additionally, the catalytic activity of the original and regenerated alumina was compared by studying the kinetics of ferulic acid pyrolysis and analyzing the obtained kinetic parameters through temperature‐programmed desorption mass spectrometry (TPD‐MS).

### 2.1 Thermogravimetric Analysis

TGA measurements were performed at a heating rate of 10°C/min, which provides a reasonable balance between experimental duration and thermal resolution. Although the heating rate can influence the apparent decomposition temperatures and mass‐loss profiles, the primary objective of this study was not to investigate the decomposition kinetics of the carbon coating in air. Instead, TGA was used to estimate the amount of carbon coating formed during pyrolysis and to evaluate the completeness of its removal after the regeneration procedure.

DTG curves (Figure 1) for the sample of ferulic acid over alumina (Fer/alumina) after pyrolysis reveal two distinct stages of weight loss at 110°C and 456°C. The first stage, occurring at 110°C, is associated with sample dehydration. The second stage, at approximately 456°C, accounts for the predominant weight loss, which is likely due to the decomposition and oxidation of the carbon coating. This finding is consistent with previous studies on Fer/alumina pyrolysis in this temperature range [41]. For the regenerated sample, most of the mass loss occurs at lower temperatures, primarily attributed to dehydration. However, minor processes detected on the DTA curve suggest the presence of a small amount of carbon in the regenerated sample. The total mass loss observed during the thermogravimetric analysis was 4.50% for the Fer/alumina sample after pyrolysis and 2.15% for the regenerated sample (Table 1).

**FIGURE 1:**
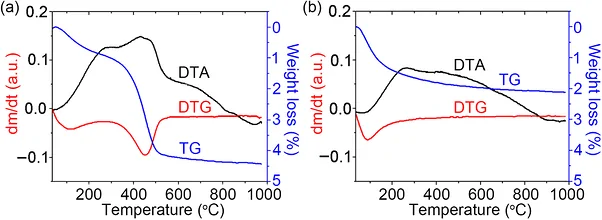
Differential thermal analysis (DTA), differential thermogravimetric analysis (DTG), and thermogravimetric (TG) curves for samples of Fer/alumina, after pyrolysis (а), and after regeneration (b).

**TABLE 1:** Thermogravimetric analysis of the alumina samples after pyrolysis ferulic acid and after regeneration.

Sample	Stage	*Т*_max_ (°C)	Weight loss (%)
Fer/alumina after pyrolysis	I	110	0.85
	II	456	3.65
	∑(I+II)	—	4.50
Regenerated alumina	I	90	2.15

### 2.2 X‐Ray Diffraction Analysis

X‐ray diffraction patterns of the initial alumina, the pyrolyzed Fer/alumina sample, and the regenerated catalyst are presented in Figure S2. In all cases, the diffraction features are characteristic of alumina and are consistent with the reference pattern reported in the JCPDS database (file no. 00‐047‐1770). No significant changes in peak positions or overall diffraction profiles were observed after pyrolysis or regeneration, indicating that the alumina framework remains structurally stable under the applied treatment conditions.

Only weak and broad diffraction features attributable to carbon‐containing species were detected. No well‐defined reflections corresponding to highly crystalline graphitic carbon were observed. This suggests that the carbon deposits formed during ferulic acid pyrolysis are predominantly disordered or poorly crystalline, which is consistent with the XPS, FTIR, and TEM results discussed below.

The close similarity between the diffraction patterns of the initial and regenerated alumina further indicates that catalyst regeneration does not induce significant changes in the bulk structure of alumina. Therefore, the decrease in catalytic activity observed after regeneration is unlikely to originate from bulk structural transformations of the oxide and is more reasonably attributed to changes in surface chemistry and the persistence of residual carbonaceous species.

### 2.3 Transmission Electron Microscopy (TEM)

Transmission electron microscopy (TEM) was used to investigate the surface morphology and nanoparticle distribution of alumina samples before pyrolysis, after ferulic acid pyrolysis, and following regeneration (Figures 2 and 3). TEM images reveal the formation of a non‐uniform, radially oriented, striped polyaromatic carbon layer on the surface of alumina nanoparticles after pyrolysis (Figure 2), which is absent in the pristine and regenerated samples.

**FIGURE 2:**
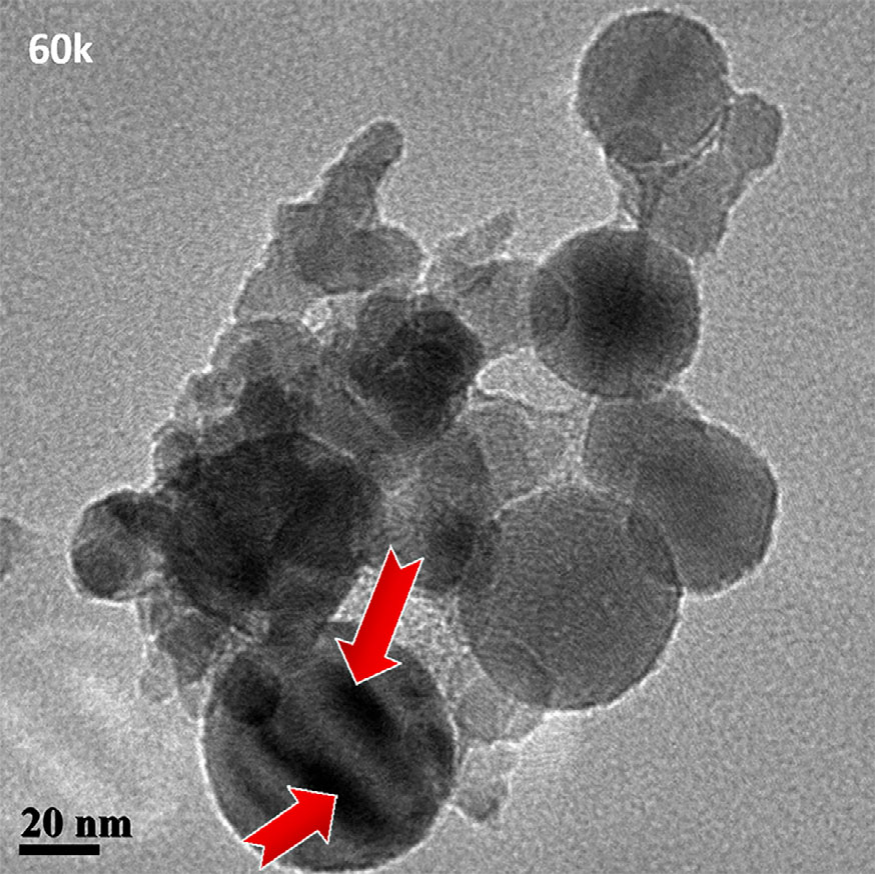
TEM images of alumina nanoparticles after ferulic acid pyrolysis, showing the formation of a radially oriented, striped polyaromatic carbon layer.

**FIGURE 3:**
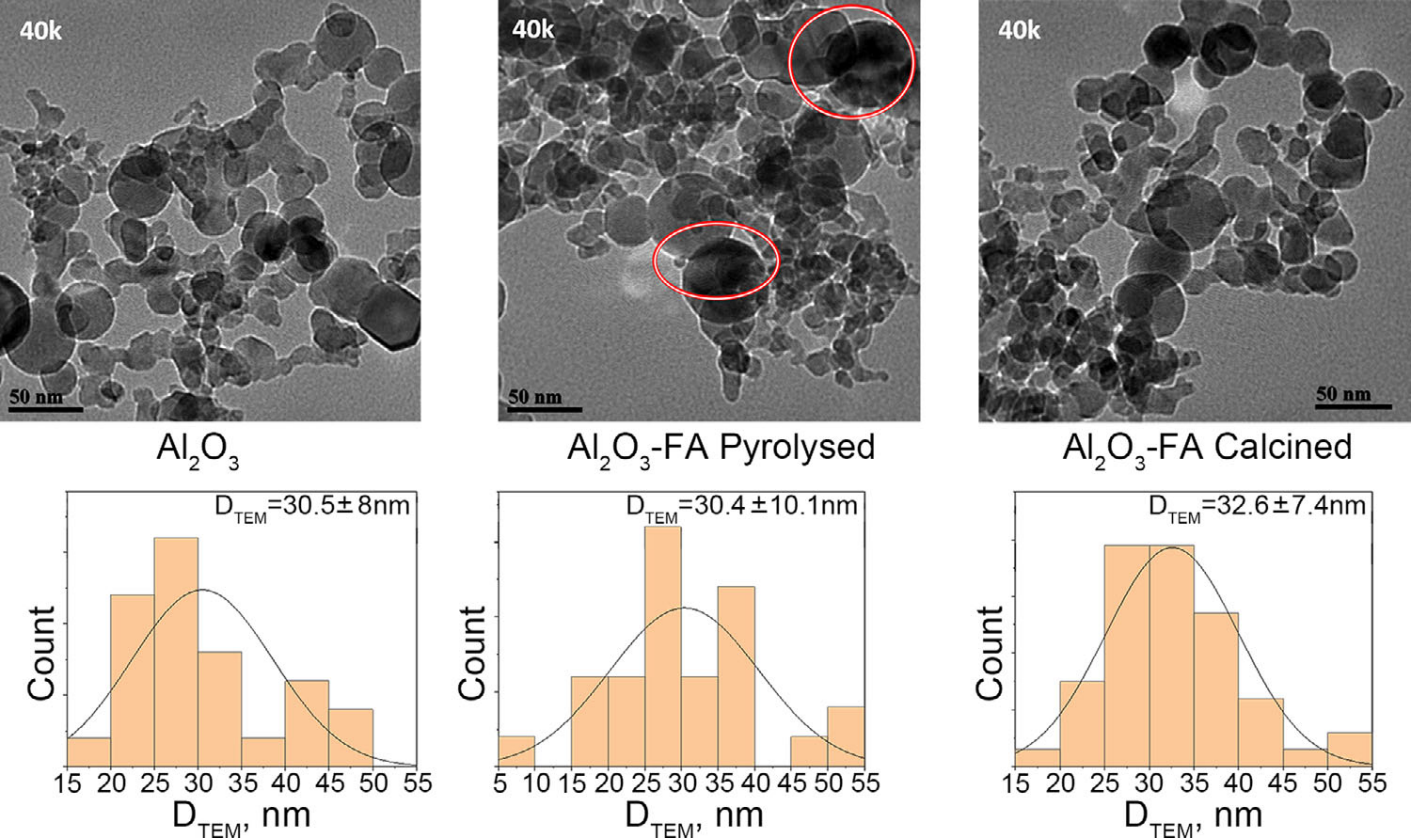
TEM images and nanoparticle size distributions of alumina samples before and after pyrolysis, and following regeneration.

Analysis of nanoparticle distributions shows that pyrolysis and subsequent regeneration induce a slight increase in the average particle size by approximately 2 nm. Although the difference remains within the experimental error range (7–10 nm), it cannot be considered statistically significant. However, this is consistent with a modest reduction in surface area of the regenerated alumina (S_Ar_ = 89 m^2^/g) compared to the initial sample (S_Ar_ = 110 m^2^/g) (Figure 3).

Similar reductions in surface area of alumina‐based materials have been widely reported and are generally attributed to thermally induced sintering, grain growth, surface diffusion, and phase transitions of transition aluminas (e.g., γ‐ to θ‐ and α‐Al_2_O_3_) under high‐temperature conditions. Recent studies further emphasize that surface area loss is governed not only by classical densification mechanisms but also by surface reorganization and defect‐driven diffusion processes occurring even in nominally dense systems [44, 45, 46].

In the present case, where the alumina is nonporous, the decrease in surface area is more appropriately associated with surface smoothing, particle coalescence, and thermally induced restructuring of external surfaces rather than pore collapse. Under such conditions, the apparent surface area loss reflects a reduction in external geometric accessibility caused by grain boundary migration, neck growth between particles, and partial surface densification during repeated thermal cycling [44, 45, 46] The overall process can be described as a thermodynamically driven minimization of surface free energy, where high‐energy surface sites are progressively eliminated under thermal stress.

The formation of the carbon layer involves topochemical solid‐phase reactions from condensed aromatic rings, resulting in the desorption of aromatics and polyaromatic hydrocarbons such as benzene, naphthalene, indene, and related compounds. These species were observed during the catalytic pyrolysis of ferulic acid (Table 2) and are consistent with prior reports on cinnamic acids, various biomass types, and their components [4, 13, 14, 15, 16, 17].

**TABLE 2:** Kinetic parameters (temperature of the maximum desorption rate *T*_max_, reaction order *n*, activation energy *E^≠^*, pre‐exponential factor *ν*_0_, and change in activation entropy *∆S^≠^*) of catalytic processes/reactions during FA pyrolysis over initial and regenerated alumina catalysts.

Pyrolysis product or Fragment ion	*m*/*z*	*T*_max_, (°C)	*n*	*E*_act_, (kJ/mol)	*ν*_0_, s^−1^	*∆S*_act_, cal/(K× mol)	R^2^
Decarboxylation							
CO_2_	44	155	1	94^a^	—	—	—
	44	250	1	111^a^	—	—	—
CO_2_ (regen.)	44	190	1	98^a^	—	—	—
	44	280	1	124^a^	—	—	—

Demethoxylation							
MeOH	31	231	1	88	3.64×10^6^	−29	0.936
	32	234	1	85	1.75×10^6^	−31	0.952
MeOH (regen.)	31	277	1	91	1.73×10^6^	−31	0.953
	32	277	1	90	1.27×10^6^	−32	0.965

Decomposition of carboxylate complexes							
4‐Vinylguaiacol (monodent.)	150	∼145 35%	1	88^a^	—	—	—
4‐Vinylguaiacol (bident.)	150	∼197 65%	1	102^a^	—	—	—
4‐Vinylguaiacol (monodent.; regen.)	150	∼193 18%	1	96^a^	—	—	—
4‐Vinylguaiacol (bident.; regen.)	150	∼242 82%	1	107^a^	—	—	—

Decomposition of carboxylate complexes with desorption of methylated products							
4‐Vinyl‐methylguaiacol	164	234	1	84	1.12×10^6^	−32	0.943
4‐Vinyl‐methylguaiacol (regen.)	164	270	1	89	1.12×10^6^	−32	0.985

Decomposition of phenolate complexes							
Guaiacol	124	234	1	84	1.12×10^6^	−32	0.943
Guaiacol (regen.)	124	∼290	1	∼94^a^	—	—	—
Phenol	94	409	1	135	8.14×10^7^	−24	0.961
Phenol (regen.)	94	473	1	143	2.29×10^7^	−27	0.931

Desorption of Aromatic/Polycyclic aromatic hydrocarbons							
Naphthalene	128	432	1	125	4.77×10^6^	−30	0.940
Naphthalene (regen.)	128	520	1	129	1.05×10^6^	−33	0.920
Indene	115	422	1	177	9.22×10^10^	−13	0.932
Indene (regen.)	115	506	1	188	1.80×10^10^	−14	0.966
Benzene	78	447	1	129	5.32×10^6^	−29	0.943
Benzene (regen.)	78	546	1	152	1.11×10^7^	−28	0.909

^a^ It was estimated approximately from equation *Е^#^* = ln(B/lnB) *RT*_max_ [31, 32, 34, 35].

The polyaromatic nature of the carbon layer observed by TEM is further supported by FTIR, XPS, and TPD‐MS analyses presented in the following sections. Based on the combined interpretation of these complementary techniques, a possible structural model of the carbonaceous layer is proposed in Scheme 2.

### 2.4 FT‐IR Spectroscopy

Analysis of the IR spectroscopy data confirms the presence of aromatic and unsaturated fragments containing C═C and C═O functional groups on the surface of the pyrolyzed sample. This conclusion is supported by the appearance of absorption bands in the corresponding regions of the IR spectrum (Figure 4, Table 3) [47, 48]. After pyrolysis of the Fer/alumina sample, almost all bands characteristic of ferulic acid immobilized on the alumina surface disappear from the IR spectrum of the pyrolyzed Fer/alumina sample [41]. Nevertheless, spectral features indicative of aromatic fragments on the surface remain clearly detectable.

**FIGURE 4:**
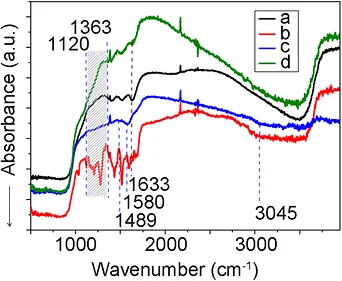
FT‐IR spectra of (a) pure alumina; (b) sample Fer/Al; (c) sample Fer/Al after pyrolysis; (d) regenerated alumina.

**TABLE 3:** Assignments of some absorbance in FT‐IR spectra (Figure 4) of pure alumina sample; Fer/alumina after pyrolysis; and regenerated alumina samples.

Absorbance (cm*^−^* ^1^)	Assignments
3200–3600	ν(O─H) stretching vibrations of various hydroxyl groups, including hydrogen‐bonded water molecules and surface hydroxyls
3045	ν(C─H_ar_) stretching vibrations of aromatic and polyaromatic compounds
1120–1363	δ(C─O─C) deformation vibrations and δ(C─H_ar_) aromatic bending vibrations
1489 and 1580	ν(C═C) stretching vibrations of the aromatic rings
1630–1750	ν(C═O) stretching vibrations

In particular, a distinct band observed near 3045 cm^−^^1^ can be assigned to aromatic C─H stretching vibrations, ν(C─H_ar_), characteristic of aromatic and polyaromatic structures. Furthermore, the absorption band centered at approximately 1580 cm^−^^1^, together with the feature around 1489 cm^−^^1^, corresponds to ν(C═C) stretching vibrations within the aromatic ring. Aromatic rings exhibit characteristic skeletal vibrations of the aromatic framework, often referred to as ring pulsation modes, which are observed as two ν(C═C) stretching vibrations in the regions of 1580–1600 and 1450–1500 cm^−^^1^ and typically appear as sharp absorption bands. The presence of conjugation within the aromatic ring leads to an increase in the intensity of these bands, accompanied by a shift toward lower wavenumbers [48]. Such spectral features, particularly the red shift, are clearly observed in the IR spectrum of the pyrolyzed sample. Carbonyl functionalities are also evidenced by the persistence of absorption in the broad region between 1630 and 1750 cm^−^^1^ [48]. In addition, a broad and complex band spanning the 3200–3600 cm^−^^1^ region is attributed to ν(O─H) stretching vibrations of various hydroxyl species, including surface hydroxyl groups and adsorbed water molecules [47, 48].

Overall, the IR spectroscopic observations are consistent with the thermal evolution of absorption bands reported for biomass samples subjected to pyrolysis at different temperatures, as described by Smith et al. [49]. In particular, the significant attenuation of C─H stretching modes near 2900 cm^−^^1^ after pyrolysis, combined with the emergence of pronounced aromatic C═C stretching vibrations near 1600 cm^−^^1^ (notably at 1580 and 1489 cm^−^^1^), indicates an increasing contribution from aromatic condensation processes during thermal treatment.

### 2.5 X‐Ray Photoelectron Spectroscopy Analysis

XPS analysis is a powerful tool for studying surface processes on catalysts, including deactivation, poisoning, coke formation, and regeneration [18, 49, 50, 51, 52, 53, 54, 55]. The XP spectra of untreated alumina reveal three carbon states, with peaks at 285.0, 286.3, and 289.1 eV, assigned to C─C/C═C, C─Hx, and C═O/C─OH, respectively, along with a carbonate‐like species (Figure 5). These peaks are typically associated with adventitious carbon, which forms from air exposure of the sample (Figure 5 and Figure S3, Table S1) [56]. Adsorption of ferulic acid introduces strong additional peaks at 284.6 and 288.5 eV. The former is indicative of aromatic ring structures, a finding supported by a weak π‐π* transition peak at 291.4 eV. The 288.5 eV peak is attributed to the carboxylic acid group (COO^−^, COOH) and its monodentate and bidentate surface complexes. Furthermore, a third O 1s peak at 533.5 eV for ferulic acid adsorbed on alumina, along with a shift in the second peak to 532 eV (Table S2), suggests the formation of ferulic acid carboxylate and phenolate complexes on the alumina surface, as previously observed in the interaction of natural phenolic compounds with metal oxide nanoparticles [57]. After pyrolysis, the intensity of the carboxylic acid functionality decreases, but the aromatic ring contribution remains strong at 284.5 eV (50%) and 291.4 eV (3%) (Table S4). An additional O 1s peak at 533.7 eV for the pyrolyzed sample and the shift of the second peak to 532.2 eV (Table S2), compared with the initial (532.5 eV) and calcined (532.6 eV) samples, may be associated with the formation of a polyaromatic layer on the alumina, as seen in the TEM images (Figures 2 and 3). After calcination, all traces of ferulic acid are removed, and the resulting C(1s) and O(1s) spectra are nearly identical to those of untreated alumina (Figure 5, Tables S1,S2). This result is consistent with the IR spectroscopy data, which show absorption in the corresponding spectral regions (Figure 4, Table 3), and aligns with findings from studies of polyphenols and miscellaneous carbon materials by other researchers [49, 50, 51, 57, 58, 59, 60, 61].

**FIGURE 5:**
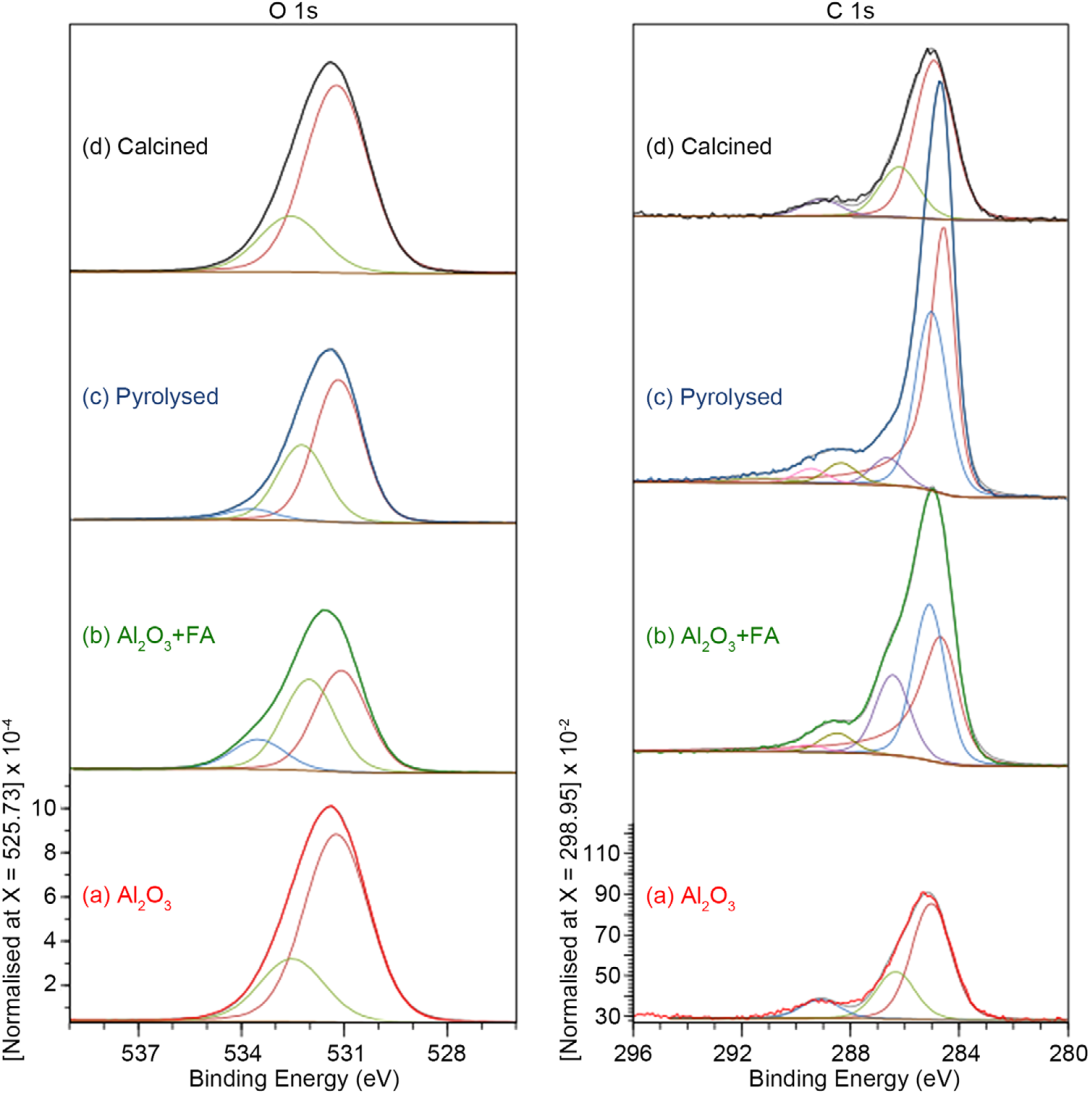
Deconvolution of O1s and C1s peaks spectra for the initial alumina sample (a), Fer/alumina sample before pyrolysis (b), Fer/alumina sample after pyrolysis (c) and after next regeneration procedure (d).

Deconvolution of the C 1s and O 1s peaks is an effective method for obtaining information about the carbon coating structure formed during the pyrolysis of biomass and its components. The deconvolution data for the C 1s and O 1s peaks from alumina samples before and after ferulic acid pyrolysis, as well as for the original and regenerated samples, are presented in Figure 5. The relative carbon and oxygen concentrations in each sample are summarized in Tables S1,S2.

Notably, deconvolution of the C 1s XPS spectrum of the pyrolyzed sample revealed six distinct components, including a characteristic π–π* shake‐up transition at 291.4 eV. The resulting C 1s peak distribution is in good agreement with both theoretical calculations and reported experimental data for substituted condensed polyaromatic hydrocarbons (e.g., pyrene, perylene, and coronene), as well as carbonaceous materials such as biochar and graphite‐like structures formed during direct and catalytic pyrolysis of second‐generation biomass [49, 50, 58, 59, 60, 61]. Importantly, the presence of the π–π* transition and the dominance of sp^2^‐hybridized carbon species are consistent with the radially oriented, striped polyaromatic carbon layer observed by TEM, supporting the formation of an extended aromatic carbon network on the alumina surface during pyrolysis.

These results support the conclusion that a polyaromatic carbon layer dominates the surface of the pyrolyzed alumina sample (Table S1). This carbon layer contains a minor fraction of oxygenated functional groups, including phenolic (Ph─O─), carbonyl (C═O), and carboxylate (O═C═O) species. Based on these XPS data, in combination with TPD–MS results discussed in Section 3.4, a plausible structural model of the polyaromatic carbon layer is proposed.

Additionally, a slight decrease in the signal intensity at 532.5 eV can be observed, which may indicate a reduction in the number of surface hydroxyl groups [52, 53], (Figure 5). This observation is consistent with the decrease in the O/Al ratio, which changes from 1.91 for the initial sample to 1.46 for the regenerated sample. This reduction can be attributed to dehydroxylation processes [53, 54, 55], and, more notably, to the blocking of catalytic hydroxyl groups due to the formation of specifically bound surface complexes.

The pyrolysis of these complexes results in the formation of residual groups that remain tightly bound to the surface, containing C═O fragments. Even subsequent regeneration fails to fully remove these residual groups from the surface (Tables S1,S2). This persistent presence of bound species is likely the primary cause of catalyst poisoning and the associated decrease in catalytic activity, which is further discussed in the TPD MS section (Table 2). Possible mechanisms for catalyst poisoning due to the formation of these specific surface complexes were proposed based on DFT calculations.

### 2.6 TPD MS Study of Alumina Catalytic Activity in Pyrolysis of Ferulic Acid

A comparative analysis of the P/T (pressure/temperature) curve for ferulic acid pyrolysis over regenerated alumina revealed notable changes in the curve shape compared to the original catalyst sample (Figure 6). Specifically, the temperature ranges for high‐temperature pyrolysis stages III and IV shifted toward higher temperatures for the regenerated catalyst by approximately 45°C–80°C, reaching 285°C and 490°C, respectively, compared to the original sample (stages: I – 60°C, II – 155°C, III – 240°C, IV – 408°C), as shown in Figure 6. These changes indicate an alteration in the temperature ranges of key pyrolysis processes such as dehydration, decarboxylation, decarbonylation, demethoxylation, and the formation of aromatic compounds. This shift is further reflected in the changes in the shape and localization of the peaks on the TPD curves for molecular and fragment ions of the main pyrolysis products: *m*/*z* 44 (CO_2_), *m*/*z* 18 (H_2_O), *m*/*z* 28 (CO), *m*/*z* 31 (CН_3_ОН), *m*/*z* 78 (C_6_Н_6_), and others, as illustrated in Figures 6 and 7.

**FIGURE 6:**
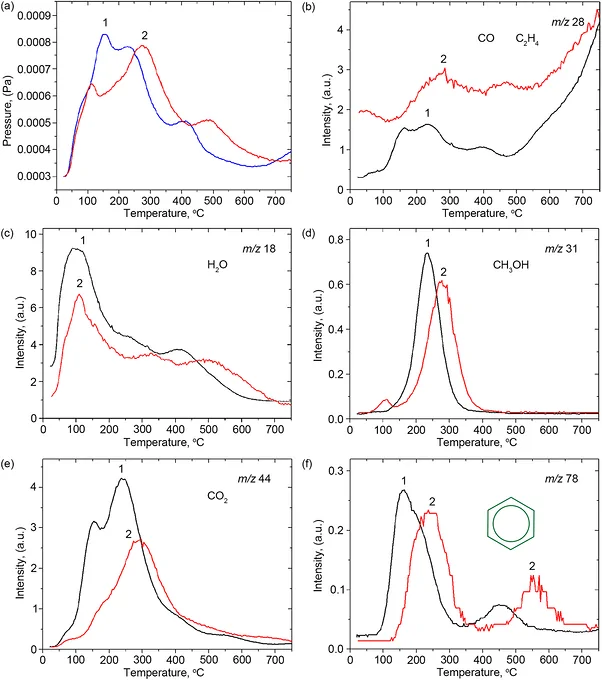
P/T curves and TPD‐curve for the ions with *m*/*z* 18 (H_2_O), *m*/*z* 28 (CO, C_2_H_4_), *m*/*z* 44 (CO_2_), *m*/*z* 31 (CH_3_OH, CH_3_O^+^) and *m*/*z* 78 (C_6_H_6_) obtained during pyrolysis of ferulic acid over the initial (1‐black curve) and regenerated (2‐red curve) alumina samples.

**FIGURE 7:**
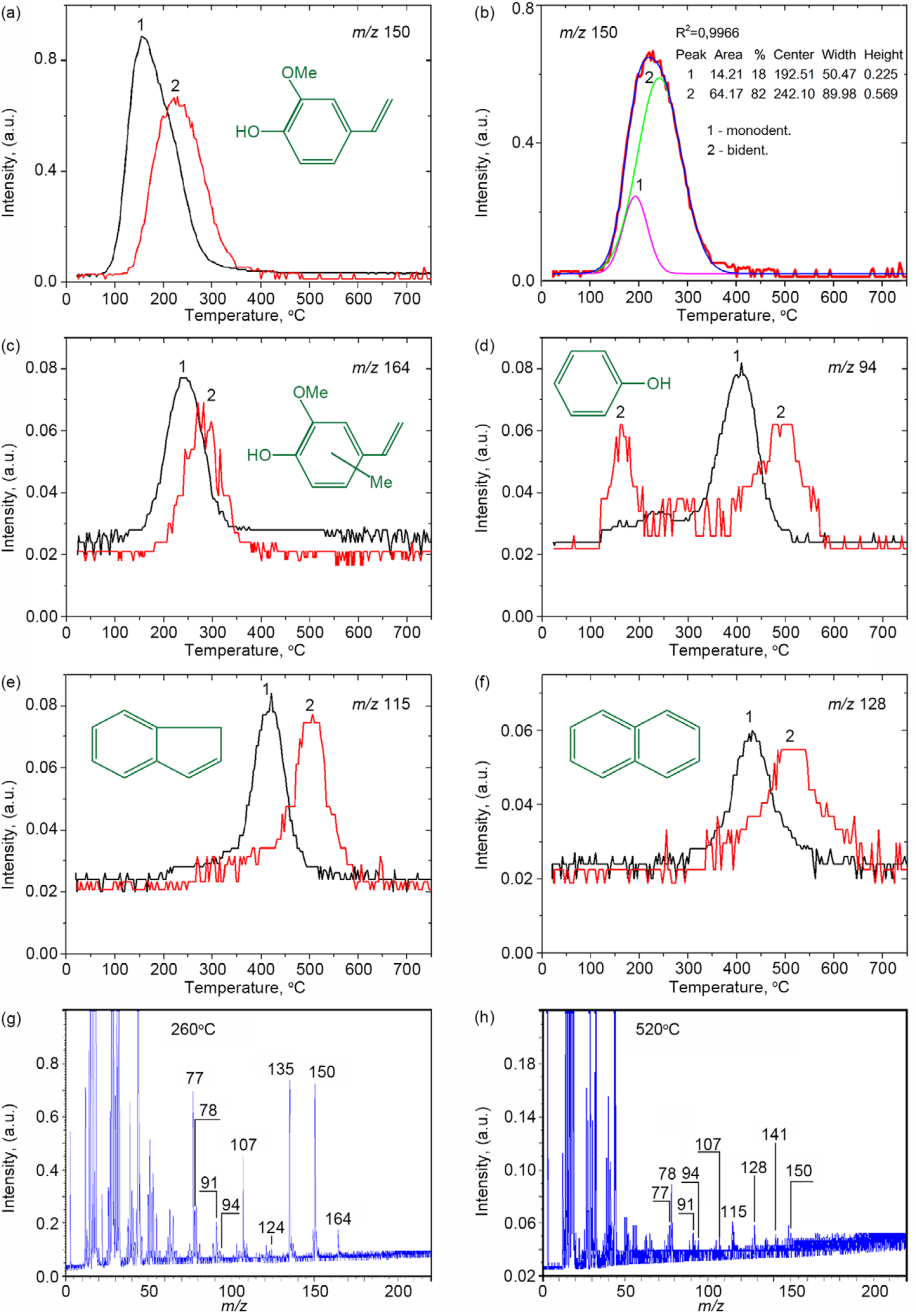
TPD‐curve for the ions with (a and b) *m*/*z* 150 (4‐vinylguaiacol), (c) *m*/*z* 164 (methylated vinyl guaiacol), (d) *m*/*z* 94 (phenol), (e) *m*/*z* 115 (indene), (f) *m*/*z* 128 (naphtalene) obtained during pyrolysis of ferulic acid over the initial (1‐black curve) and regenerated (2‐red curve) alumina samples. (b) Deconvolution of the TPD‐curve for the molecular ion of 4‐vinylguaiacol with *m/z* 150 into separate Gaussians: curve 1 – monodentate complexes and curve 2 – bidentate carboxylates. Mass spectra of pyrolysis products at 260°C (g) and 520°C (h).

At the same time, the intensity of stage II on the P/T curve decreased for the regenerated sample, which correlates with the reduced intensity of the peak at 150°C on the TPD curve for *m*/*z* 44 (CO_2_), indicating a decrease in decarboxylation processes. Furthermore, the *T*_max_ of the high‐temperature decarboxylation stage shifted by approximately 50°C to higher temperatures, reaching 280°C (Figure 6, Table 2). As established in our previous studies, the decarboxylation of cinnamic acids occurs through thermal transformations of mono‐dentate and bidentate chelate carboxylate complexes, leading to the formation of corresponding substituted vinylbenzenes. In the case of ferulic acid, this results in the formation of 4‐vinyl guaiacol (*m/z* 150, 135, 107, etc.), (Figure 7a,b,g) [15, 41, 62, 63, 64]. This shift is evident in the high‐temperature region for the *T*_max_ of the peak corresponding to the 4‐vinyl guaiacol molecular ion on the TPD curve (Figure 7a,b,g, and Table 2). Specifically, the shift is approximately ∆*T*_max_ = 225°C – 160°C ≈ 65°C, corresponding to an increase in the activation energy for 4‐vinyl guaiacol formation by ∆*E*_act_ ≈ 14 kJ/mol.

As shown in our previous work [41], the desorption peak for 4‐vinyl guaiacol results from the superposition of two distinct peaks at *T*_max_ = 145°C and *T*_max_ = 197°C, corresponding to the decomposition of mono‐dentate and bidentate chelate carboxylate complexes, respectively (Scheme 1). To facilitate the mechanistic interpretation, Scheme 1 summarizes the reaction pathway for 4‐vinylguaiacol formation via Mechanism B and includes the identified prereaction complexes, intermediates, and transition states obtained from the DFT study (see Section 2.5).

**SCHEME 1:**
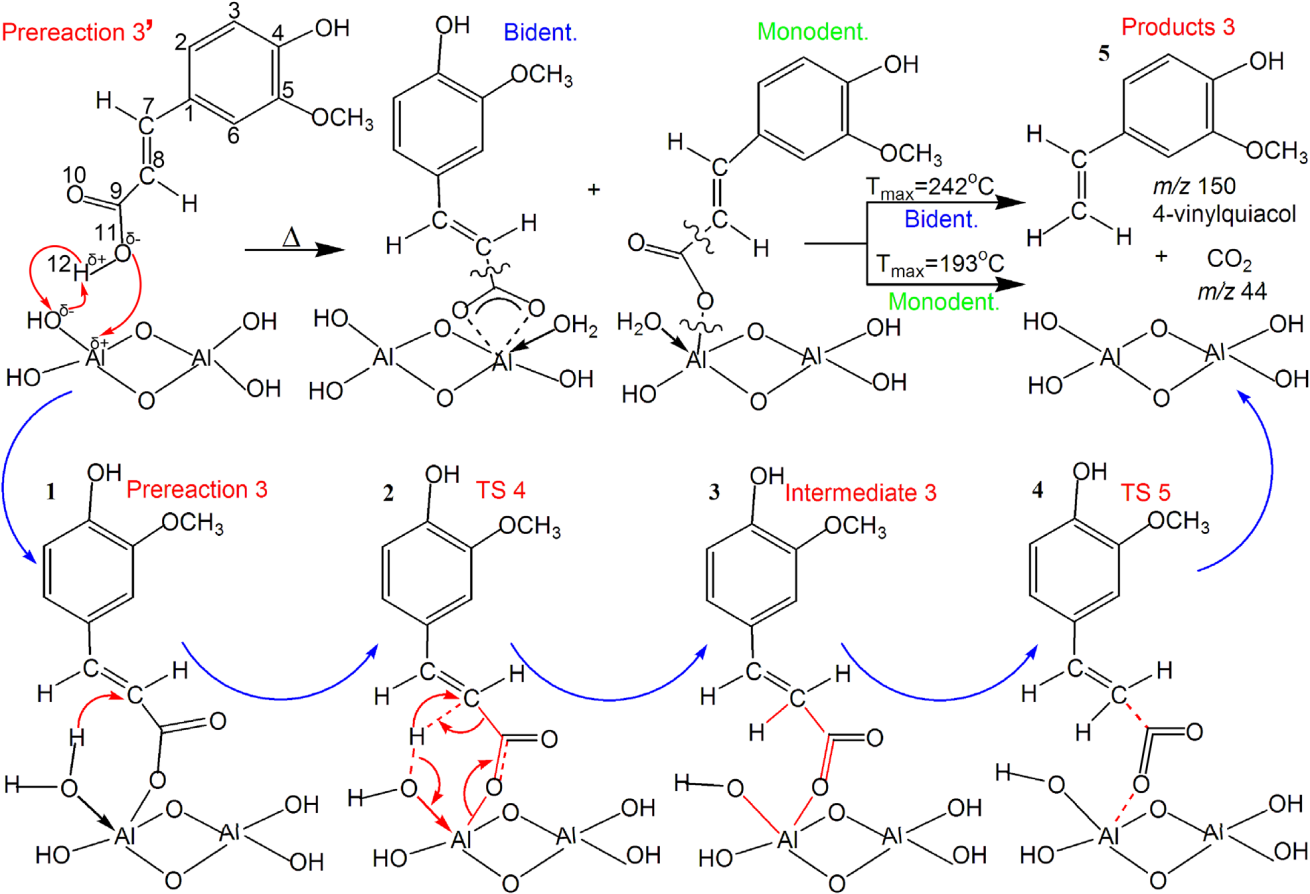
Proposed mechanism for the formation of 4‐vinylguaiacol via the decomposition of monodentate and bidentate chelate carboxylates on the alumina surface, according to Mechanism B derived from DFT calculations (see Section 2.5).

Deconvolution of the TPD curve peak for *m*/*z* 150 in the regenerated sample revealed two separate peaks at *T*_max_ = 193°C and *T*_max_ = 242°C, Figure 7b. Notably, the proportion of mono‐dentate complexes, which decompose at the lower temperature, decreased nearly twofold to 18%, compared to 35% for the original catalyst sample [41]. This decrease is likely due to a reduction in the fraction of active sites caused by catalyst poisoning and dehydroxylation.

These changes are evidently associated with a reduction in the proportion of highly reactive active sites on the surface of the regenerated catalyst. This is primarily due to dehydration and dehydroxylation processes, as indicated by the decreased intensity of water desorption in the 60°C–250°C range. Additionally, a broadening of the water desorption temperature range—extending up to ∼700 °C for the regenerated sample compared to ∼600 °C for the original sample—further supports the occurrence of dehydroxylation of surface hydroxyl groups (Figure 6). These effects are compounded by the chemical poisoning of active sites, likely due to the formation of tightly bound C═O‐containing species that cannot be removed under the applied regeneration conditions, as evidenced by XPS analysis.

In addition to the observed shift of decarboxylation and dehydration processes toward higher temperatures, a similar trend is evident for demethoxylation reactions (*m*/*z* 32, 31), the formation of methylated derivatives of 4‐vinyl guaiacol (*m*/*z* 164), phenol (*m*/*z* 94), and various aromatic and condensed aromatic compounds such as benzene (*m*/*z* 78), indene (*m*/*z* 115), and naphthalene (*m*/*z* 128) (Figure 7, Scheme 2, Table 2).

SCHEME 2Proposed surface pathway for styrene‐like polymerization of ferulic acid phenolate complexes on alumina (Stage I), followed by thermal decomposition of the resulting polymeric layer (Stage II), leading to the formation of extended polyaromatic carbon domains. Formation and release of phenol (*m*/*z* 94), indene (*m*/*z* 116, 115), naphthalene (*m*/*z* 128), and benzene (*m*/*z* 78) detected by TPD–MS.

**Figure:**
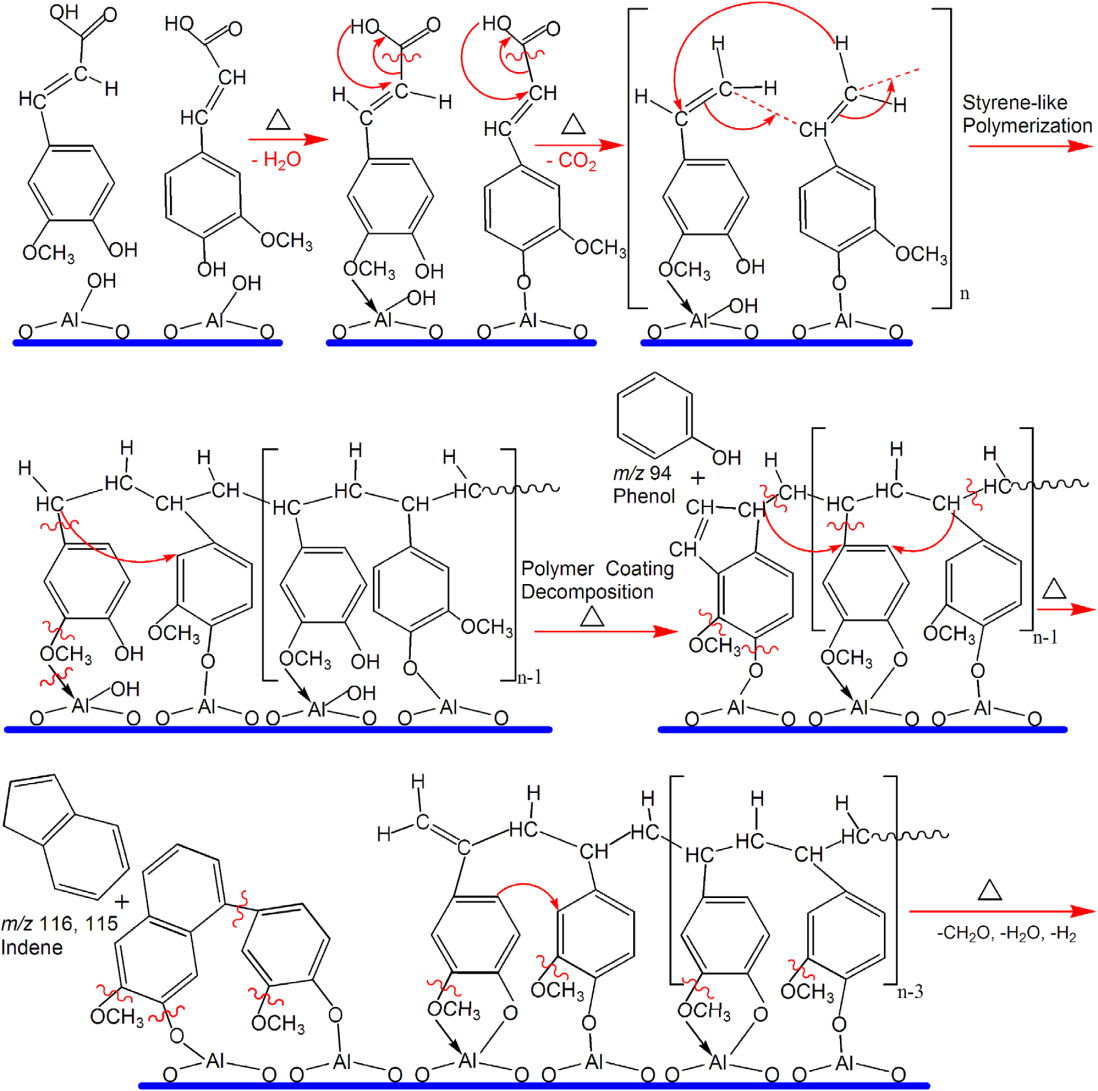

**Figure:**
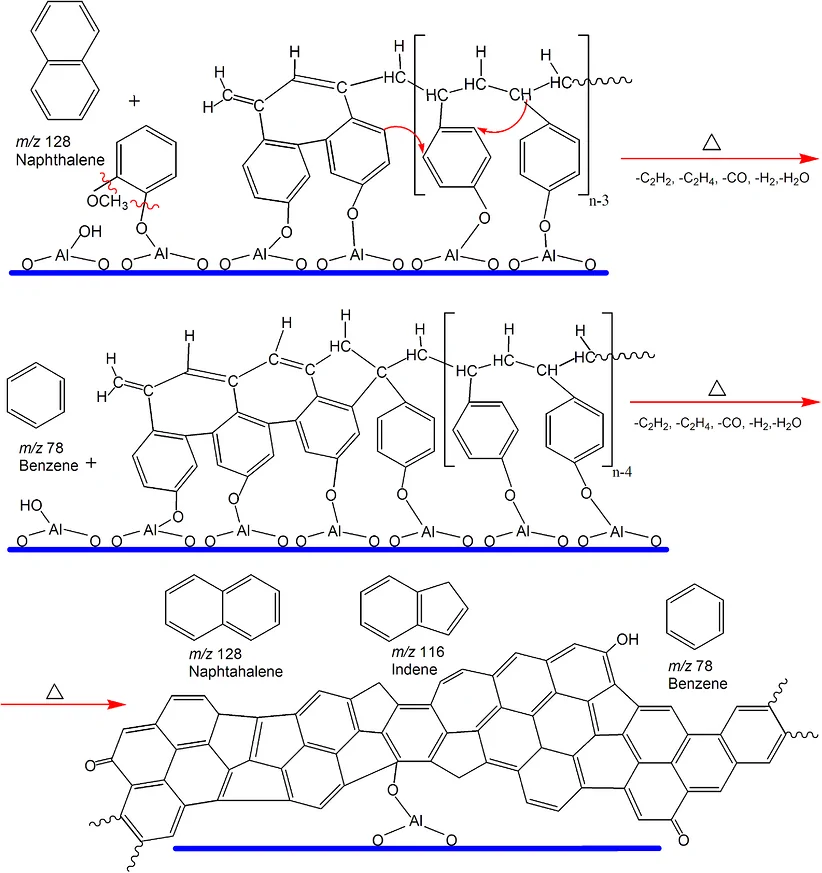

Kinetic parameter calculations confirm that the temperature of the maximum reaction rate for the formation of these catalytic products shifted to higher values by approximately 30°C–100°C, accompanied by a corresponding increase in activation energies (Table 2). The most pronounced changes were observed for high‐temperature transformation processes, particularly in the formation of phenol and benzene. Notably, the temperature shift for benzene formation was approximately ∆*T*_max_ = 546 – 447≈100°C, with an associated increase in activation energy of ∆*E*_act_ ≈ 23 kJ/mol.

Based on the combined XPS, FTIR, TEM, and TPD–MS results, a structural model of the polyaromatic carbon layer formed on the alumina surface is proposed (Scheme 2). Although the detailed elementary steps of carbon domain formation cannot be resolved directly due to the complexity of the topochemical transformations occurring on the alumina surface, the pathway proposed in Scheme 2 is supported by several complementary experimental observations. In particular, the evolution of aromatic products detected by TPD–MS, the changes observed in the FTIR spectra, the appearance of the π–π* shake‐up feature in the C 1s XPS spectrum, and the non‐uniform, radially oriented, striped polyaromatic carbon layer observed on the surface of alumina nanoparticles by TEM after pyrolysis collectively support the formation of extended polyaromatic domains during pyrolysis.

As illustrated in Scheme 2, the process is initiated by the polymerization of ferulic acid phenolate complexes, proceeding through a styrene‐like polymerization pathway and leading to the formation of a polymeric surface layer accompanied by CO_2_ release. Upon further heating (Stage II), this polymeric layer undergoes thermal decomposition, resulting in the evolution of various aromatic products detected by TPD–MS (Figures 6 and 7) and the gradual development of extended polyaromatic carbon domains on the alumina surface.

Therefore, the pathway presented in Scheme 2 should be regarded as a mechanistically informed proposal based on the combined TPD–MS, FTIR, XPS, and TEM results. Importantly, the sequence of the proposed transformations is consistent with the order of aromatic product evolution observed during TPD–MS analysis. For both the initial and regenerated alumina samples, phenol is released at the lowest temperatures, followed by indene, naphthalene, and finally benzene. For the initial alumina sample, the desorption maxima increase in the order phenol (409°C) < indene (422°C) < naphthalene (432°C) < benzene (447°C), while for the regenerated sample the corresponding sequence is phenol (473°C) < indene (506°C) < naphthalene (520°C) < benzene (546°C).

This systematic increase in *T*_max_ suggests a progressive transformation of oxygen‐containing aromatic species into increasingly condensed and thermally stable polyaromatic structures. Within this framework, phenol may be regarded as a product originating from oxygenated aromatic moieties, whereas indene and naphthalene are indicative of progressively more condensed aromatic structures. The release of benzene at the highest temperatures is consistent with the thermal decomposition of the most stable polyaromatic carbon domains. Collectively, these observations support the proposed sequence of condensation, aromatization, and carbonization processes leading to the formation of extended polyaromatic carbon domains represented in Scheme 2.

This transformation is likely more complex than depicted in Scheme 2 and may involve the formation and subsequent breakdown of a polystyrene‐like intermediate, followed by structural reorganization into condensed aromatic systems. In parallel, a variety of secondary thermal reactions, including electrocyclization, cycloaddition, Diels–Alder, and other pericyclic processes, may contribute to the evolution of the carbonaceous network. Such processes are further facilitated by pyrolysis‐generated reactive unsaturated species, including CO (*m*/*z* 28), C_2_H_2_ (*m*/*z* 26), C_2_H_4_ (*m*/*z* 28), H_2_C═C═O (*m*/*z* 42), molecular hydrogen, as well as ketene‐ and quinonoid‐type surface intermediates. In addition, atomic hydrogen and radical species generated from these reactive intermediates can undergo surface diffusion and spillover processes [65], thereby promoting bond rearrangement, deoxygenation, and restructuring of the growing carbonaceous layer.

In line with this mechanistic picture, XPS analysis reveals a π–π* shake‐up contribution of approximately 3% in the C 1s spectrum, indicating the presence of extended aromatic conjugation within the polyaromatic carbon layer. This interpretation is further supported by IR spectroscopy, which shows pronounced aromatic C═C stretching vibrations in the 1600 cm^−^^1^ region (notably at 1580 and 1489 cm^−^^1^), consistent with progressive aromatic condensation.

These spectroscopic features are in good agreement with the radially oriented, striped polyaromatic carbon layer observed by TEM (Figures 2 and 3). In particular, TEM images of the pyrolyzed sample (Figure 1, upper right panel) reveal elongated neck‐ and filament‐like structures, approximately 1–2 nm in width and up to 40–50 nm in length, bridging alumina particles or extending across their surfaces. These features may arise from either neck formation between adjacent alumina particles or the development of elongated polyaromatic carbon structures during pyrolysis.

Such surface migration and spillover‐assisted growth processes may account for the formation of banded or striped coating patterns on spherical alumina particles, without implying long‐range graphitic ordering. The resulting conjugated aromatic domains facilitate electron delocalization and may therefore contribute to catalytic activity in transformations of phenolic biomass‐derived compounds, in agreement with DFT studies reported by Verma et al. [21].

### 2.7 Quantum Chemical Calculations

To understand the nature of the decrease in the catalytic activity of alumina and establish the mechanisms of the decarboxylation reaction, DFT calculations were performed on the thermal transformations of carboxylate complexes of ferulic acid. Considering the fact that decarboxylation is one of the most critical processes for the catalytic conversion of lignin‐rich waste biomass because it allows obtaining such industrially important monomers as renewable styrenes and, in particular, 4‐vinyl guaiacol [36, 37, 38, 39, 40, 43].

#### 2.7.1 Decarboxylation of Ferulic Acid in the Gas Phase

As a reference system, the decarboxylation of ferulic acid was first investigated in the gas phase (vacuum). The molecular structure and atom numbering used throughout the mechanistic discussion are shown in Scheme 1 and Figure S8a. The initial geometry was adopted from the literature [66].

The calculated reaction pathway, including the reactant, transition state (TS1), and products, is presented in Figure 8, while the corresponding relative Gibbs free energy profile is shown in Figure 8A. The reaction proceeds through a single concerted decarboxylation step involving proton transfer from the carboxyl group to the adjacent C8 carbon atom. This proton‐transfer event is accompanied by cleavage of the C8─C9 bond and release of carbon dioxide.

FIGURE 8(A) Relative Gibbs free energy profiles (Δ*G*, kJ mol^−^^1^) for ferulic acid decarboxylation in the gas phase and on alumina via the proposed acidic pathways. All reaction profiles are referenced to the corresponding prereaction complex (Δ*G* = 0 kJ mol^−^^1^). Transition states (TS) and intermediates (Int) are reported as relative Gibbs free energies (Δ*G* ^‡^ and Δ*G*, respectively). The black profile corresponds to gas‐phase decarboxylation, the blue profile to Mechanism A (surface‐assisted acidic decarboxylation via weakly coordinated adsorption), the pink profile to Mechanism B (acidic decarboxylation via dissociative adsorption and formation of a grafted surface carboxylate), and the red profile to Mechanism C (surface “stitching” pathway leading to catalyst poisoning and subsequent formation of guaiacol‐containing products). Abbreviations of stationary points are defined in Section 2.5. (B) Optimized structures of the key stationary points associated with the reaction pathways shown in panel A: (a) gas‐phase decarboxylation of ferulic acid; (b) Mechanism A, including the prereaction complex, transition states, intermediate, and products; (c) Mechanism B, including Prereaction3′, Prereaction3, transition states, intermediate, and products; and (d) Mechanism C, including the prereaction complex, transition states, Products4 formed through surface “stitching”, and Products5 generated during subsequent decomposition of Products4. Structures are oriented to maximize visibility of the reactive region and the bond‐forming/bond‐breaking events.

**Figure:**
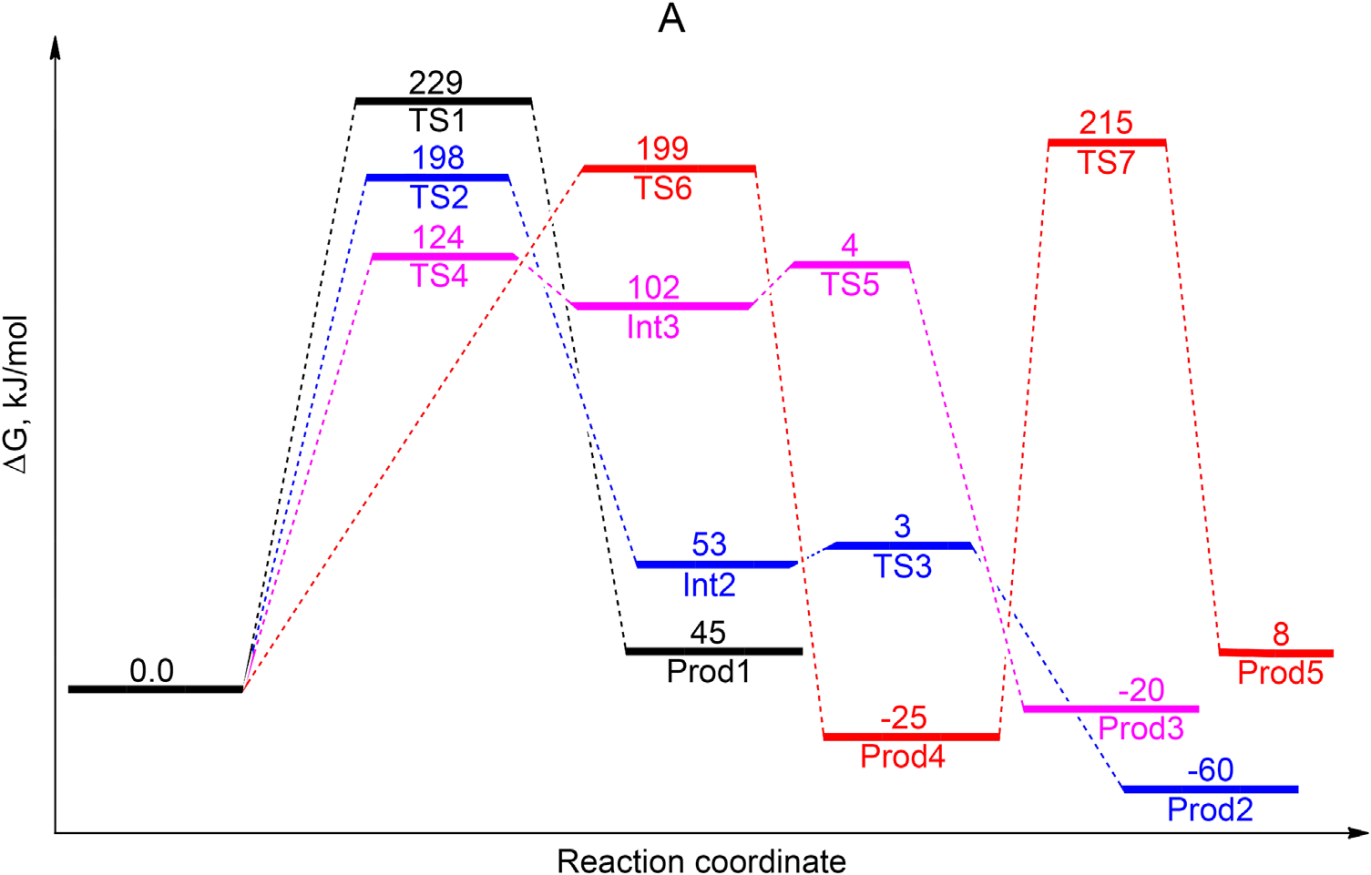

**Figure:**
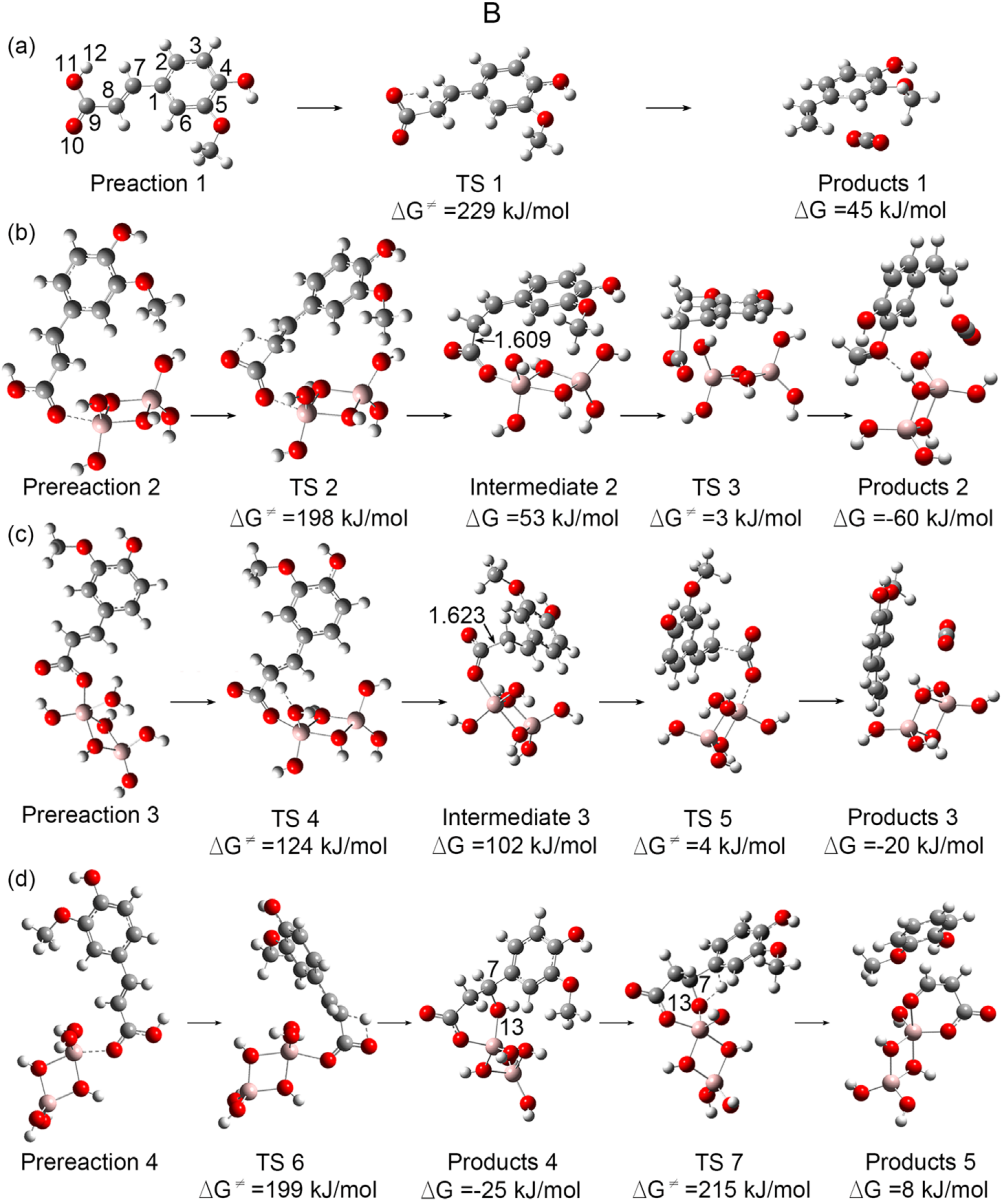

The nature of TS1 was verified by intrinsic reaction coordinate (IRC) calculations, which confirmed that the transition state directly connects the reactant and product minima on the potential energy surface (Figure S4). The reaction yields 4‐vinylguaiacol and CO_2_, which remain associated as a weakly bound van der Waals complex immediately after decarboxylation.

The gas‐phase pathway serves as a reference for evaluating the catalytic role of alumina in the surface‐assisted mechanisms discussed below. Comparison of the calculated energy profiles demonstrates that interaction with the alumina surface substantially alters the reaction energetics and opens alternative reaction channels that are not accessible in the gas phase.

The calculated activation free energy for gas‐phase decarboxylation (Δ*G* ^‡^ = 229 kJ mol^−^^1^) is substantially higher than that obtained for the most favorable surface‐assisted pathway (Mechanism B). This result highlights the catalytic role of alumina in facilitating ferulic acid decarboxylation and promoting the selective formation of 4‐vinylguaiacol. Moreover, the gas‐phase reaction proceeds through a single concerted pathway, whereas interaction with the alumina surface enables alternative adsorption modes and reaction channels that significantly modify the reaction energetics and mechanism.

#### 2.7.2 Decomposition of Ferulic Acid on the Alumina

##### 2.7.2.1 General Considerations and Energy Reference Framework

The catalytic transformation of ferulic acid on alumina was investigated via density functional theory (DFT) calculations considering multiple mechanistic scenarios consistent with the nature of the alumina surface. All Gibbs free energy profiles are reported as relative values (Δ*G*, kJ mol^−^^1^), referenced to the corresponding prereaction adsorption complex, which is defined as Δ*G* = 0. Transition states (TS) and intermediates (Int) are expressed as Δ*G* ^‡^ and Δ*G*, respectively. This representation enables direct comparison of energetic trends across mechanistic pathways without reliance on absolute energy values.

Before discussing the individual reaction pathways, it should be emphasized that the nature of alumina provides several types of catalytically active sites. As shown previously [29], the alumina surface contains both Lewis acidic aluminum centers and basic oxygen/hydroxyl sites, enabling ferulic acid to form different adsorption complexes depending on its interaction mode with the surface.

Accordingly, the most plausible mechanistic scenarios consistent with the surface chemistry of alumina were considered in this work. Mechanism A involves decarboxylation mediated predominantly by acidic surface sites, Mechanism B proceeds through a grafted surface carboxylate species, and Mechanism C represents a cooperative acid–base pathway. In addition, two additional pathways were also explored: Mechanism D, associated primarily with basic surface sites, and an alternative acid–base route (Mechanism F), both of which may lead to catalyst deactivation.

Overall, the set of mechanisms considered reflects the most likely reaction possibilities dictated by the amphoteric nature of the alumina surface. Rather than exhaustively enumerating all conceivable routes, the present study focuses on representative pathways supported by experimentally observed adsorption complexes and their corresponding computational models [41].

The role of the alumina surface in the reaction mechanism is essential and cannot be considered negligible. In all investigated mechanisms, the carboxyl group of ferulic acid remains directly coordinated to a surface aluminum center throughout the prereaction complexes, transition states, and intermediates. For Mechanisms A and C, the carbonyl oxygen interacts with aluminum via a coordination bond, whereas in Mechanism B the carboxylate group forms a more strongly bound Al─O surface linkage associated with dissociative adsorption.

These surface interactions significantly constrain the molecular geometry of the adsorbed species, reduce the number of accessible degrees of freedom, and thereby facilitate the decarboxylation process. Importantly, comparison with the corresponding gas‐phase reactions reveals that the presence of alumina lowers the activation barrier by approximately 30 kJ mol^−^^1^ for Mechanism A and by about 100 kJ mol^−^^1^ for Mechanism B (Figure 8).

Thus, although some elementary steps may appear formally concerted, the catalyst actively participates in the reaction through adsorption, stabilization of reactive configurations, and substantial modification of the reaction energetics.

##### 2.7.2.2 Decomposition of Ferulic Acid on Alumina via Mechanism A

In the first stage, the adsorption structure of ferulic acid on an Al_2_(OH)_6_ cluster was localized, in which the molecule remains largely undissociated and interacts weakly with the surface. This state is defined as the prereaction complex for Mechanism A. The corresponding structures of the initial adsorbed state, intermediates, transition states, and products are shown in Figure 8Bb, while the relative Gibbs free energy profile is presented in Figure 8A.

The prereaction structure corresponds to a monodentate adsorption mode via the carbonyl oxygen coordinated to a surface aluminum center. This interaction orients the carboxyl proton in proximity to the reactive carbon center (C8), facilitating intramolecular proton transfer in the transition state TS2. The mechanistic validity of this pathway was confirmed by IRC calculations, which connect TS2 with Intermediate2 (Int2) and the final products (Prod2).

Intermediate2 exhibits a significantly elongated C8─C9 bond (1.609 Å; bond order 0.510), indicating partial bond weakening prior to decarboxylation. Further transformation proceeds via TS3, leading to the formation of CO_2_ and 4‐vinylguaiacol (Products2). The first decarboxylation step is rate‐determining within this pathway.

The calculated relative Gibbs free energy barrier (Δ*G* ^‡^ = 198 kJ mol^−1^) indicates that this mechanism is energetically accessible but less favorable compared with alternative surface‐assisted pathways, which motivated the consideration of additional mechanisms.

##### 2.7.2.3 Decomposition of Ferulic Acid on Alumina via Mechanism B (Dominant Pathway)

Mechanism B involves dissociative adsorption of ferulic acid on the alumina surface, leading to formation of a chemisorbed carboxylate species. The process begins with the formation of a monodentate adsorption complex from a molecular complex (Prereaction3’), followed by proton transfer to a surface hydroxyl group and generation of a coordinated water molecule (Scheme 1). This step yields a grafted surface carboxylate species (Prereaction3), which represents the true starting point of the catalytic cycle (Figure 8A,Bc).

To improve mechanistic clarity, the full pathway is explicitly resolved in Scheme 1, including all prereaction complexes, intermediates, and transition states. The species Prereaction3 should be regarded as a surface‐bound intermediate rather than a free ferulic acid molecule, consistent with previous experimental and computational evidence [41].

The subsequent decarboxylation proceeds via TS4, where a coordinated water molecule mediates proton transfer to the C8 carbon atom through a six‐membered cyclic transition state (Scheme 1). This step represents the rate‐determining event of Mechanism B and accounts for the overall activation barrier of the reaction. Notably, this pathway exhibits the lowest activation free energy for the rate‐determining step among all investigated mechanisms (A–F), highlighting its unique kinetic favorability. This finding is further consistent with the experimentally observed evolution of 4‐vinylguaiacol at relatively low T_max_ values in TPD–MS experiments, supporting the proposed catalytic role of alumina in lowering the effective energy barrier. IRC analysis confirms the connectivity between TS4 and Intermediate3 (Int3), characterized by a weakened C8─C9 bond (1.623 Å; bond order 0.349) (Figures 8A,Bc). Further evolution leads to CO_2_ release and formation of 4‐vinylguaiacol.

This pathway exhibits a significantly lower relative Gibbs free energy barrier (Δ*G* ^‡^ = 124 kJ mol^−^^1^), making it the most favorable among the investigated mechanisms (Figures 8).

##### 2.7.2.4 Surface “Stitching” Pathway and Catalyst Deactivation (Mechanism C)

In addition to the productive decarboxylation pathways discussed above, an alternative reaction channel (Mechanism C) was identified that may contribute to catalyst deactivation. The prereaction complex (Prereaction4, Figure 8Bd) is structurally similar to that considered for Mechanism A, involving monodentate coordination of ferulic acid to a surface aluminum center. However, the subsequent transformation proceeds through a fundamentally different reaction pathway.

Although the transition state TS6 resembles TS2 geometrically, IRC analysis revealed that it does not lead to direct decarboxylation. Instead, following proton transfer from the carboxyl group to the C8 carbon atom, the activated ferulic acid fragment undergoes covalent attachment (“stitching”) to the alumina surface through formation of a C7─O13 bond involving a surface hydroxyl group. The resulting surface‐bound intermediate, Products4 (Prod4), therefore differs fundamentally from Products2 (Prod2) obtained via Mechanism A.

The proposed surface stitching is supported by bond‐order analysis. In Products4, the C7─O13 bond exhibits a bond order of 0.513, while the Al─O13 bond remains strong (bond order 0.780), indicating stable anchoring of the organic fragment to the alumina surface. Consequently, Products4 cannot be regarded as a decarboxylation product but rather as a surface‐trapped intermediate that may undergo subsequent transformations.

Further decomposition of Products4 was therefore investigated (Figure 8Bd). One of the products formed through this pathway is guaiacol, experimentally detected at *T*_max_ ≈ 234°C (Table 2). Accordingly, the Gibbs free energies for this reaction sequence were evaluated at the corresponding temperature (Figure 8A).

Importantly, the final state Products5 (Prod5) does not regenerate a free alumina surface. Residual organic fragments remain chemically attached to the catalyst, suggesting that Mechanism C may represent a molecular‐level pathway for catalyst poisoning and progressive carbonaceous layer formation. This interpretation is consistent with the experimental observation of persistent carbon deposits on regenerated alumina and with the development of polyaromatic carbon structures discussed in Scheme 2.

##### 2.7.2.5 Base‐Catalyzed Pathway on Alumina (Mechanism D)

In addition to the acidic pathways discussed above, a base‐catalyzed mechanism (Mechanism D) was also considered because alumina possesses not only Lewis acidic aluminum centers but also basic hydroxyl and oxygen sites capable of interacting with ferulic acid.

In this mechanism, adsorption proceeds through formation of a bidentate ferulate surface complex, Prereaction5, (Figure 9Ba), in which the carboxylate group coordinates to an aluminum center. Unlike Mechanism B, the water molecule generated during dissociative adsorption is not retained within the aluminum coordination sphere but remains associated with neighboring surface hydroxyl groups. This creates the possibility for subsequent nucleophilic reactions involving water‐derived hydroxyl species.

**FIGURE 9:**
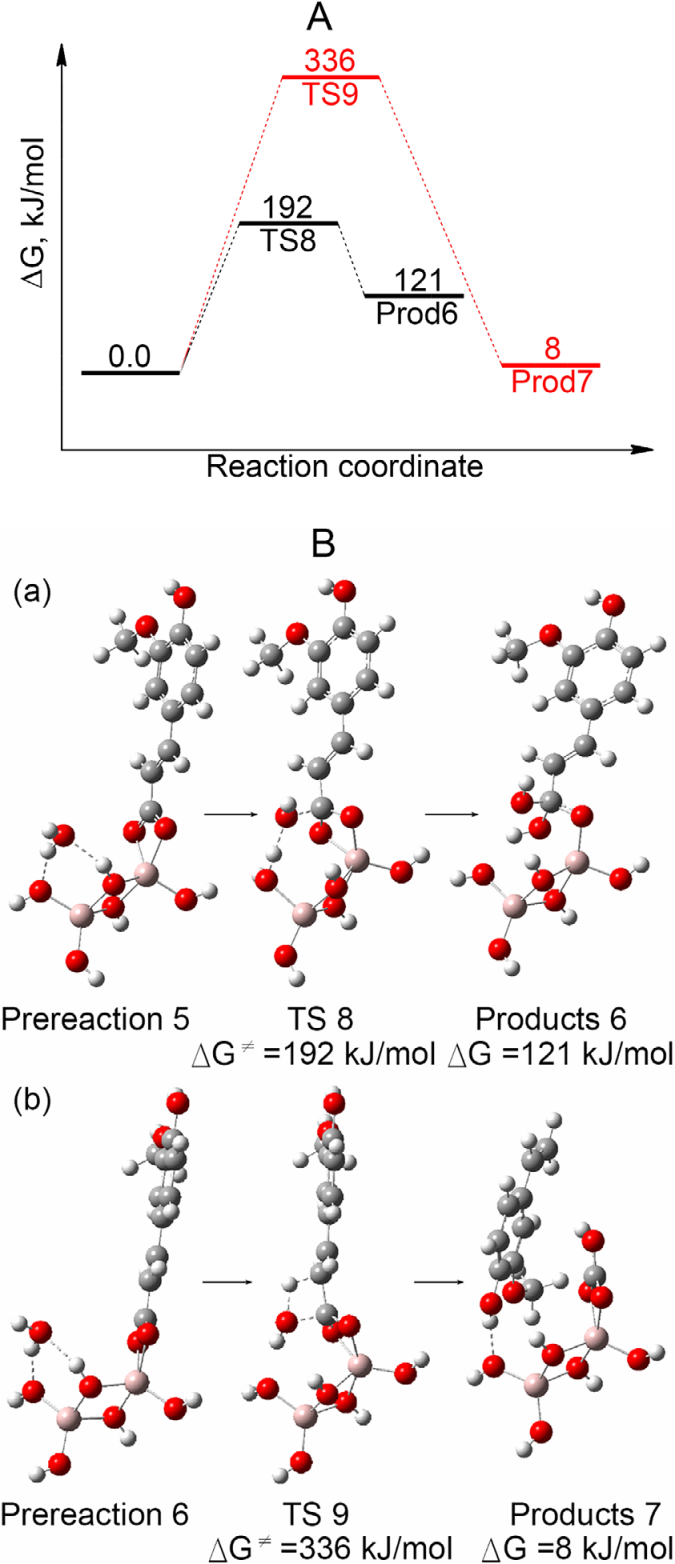
(A) Relative Gibbs free energy profiles (Δ*G*, kJ mol^−^^1^) for ferulic acid transformation on alumina via the proposed basic and acid–base catalytic pathways. All reaction profiles are referenced to the corresponding prereaction complex (Δ*G* = 0 kJ mol^−^^1^). Transition states (TS) and intermediates are shown as relative Gibbs free energies (Δ*G* ^‡^ and Δ*G*, respectively). The black profile corresponds to Mechanism D (base‐catalyzed pathway involving nucleophilic attack by surface‐generated hydroxyl species and formation of surface‐bound intermediates), whereas the red profile corresponds to Mechanism F (concerted acid–base pathway involving water‐assisted proton transfer and decarboxylation). Abbreviations of stationary points are defined in Section 2.5. (B) Optimized structures of the key stationary points associated with the reaction pathways shown in panel A: (a) Mechanism D, including the prereaction complex, transition state, and surface‐bound intermediate formed by nucleophilic attack at the carboxyl group; and (b) Mechanism F, including the prereaction complex, transition state, and products formed through the concerted acid–base decarboxylation pathway. Structures are oriented to maximize visibility of the reactive region and the bond‐forming/bond‐breaking events.

The key step of Mechanism D proceeds through transition state TS8 and involves nucleophilic attack of a hydroxyl group on the carboxyl carbon atom (C9), accompanied by proton transfer between adjacent surface hydroxyl groups. IRC analysis confirmed that this pathway leads to formation of Products6 (Prod6), a surface‐bound intermediate containing a nonclassical dicarboxylic‐acid‐type fragment coordinated to alumina through a carboxylate oxygen atom.

Comparison of the relative Gibbs free energies of Prereaction5 and Prod6 (Figure 9A) indicates that the resulting intermediate is thermodynamically stable and is therefore more likely to serve as a precursor for subsequent surface transformations rather than as a direct source of volatile reaction products. Consequently, Mechanism D appears less favorable than the acidic decarboxylation pathway (Mechanism B) and may contribute primarily to the formation of persistent surface‐bound species.

##### 2.7.2.6 Concerted Acid–Base Pathway (Mechanism F)

As discussed for Mechanism D, dissociative adsorption of ferulic acid may generate water molecules that remain mobile within the surface hydroxyl network. Such species can participate in additional acid–base transformations and therefore provide a basis for considering a concerted acid–base pathway (Mechanism F).

In this mechanism (Figure 9A,Bb), a water molecule simultaneously interacts with the C8 and C9 atoms of the activated carboxyl side chain. The corresponding transition state (TS9) involves a concerted process in which a proton is transferred to the C8 carbon atom while the hydroxyl group attacks the C9 carboxyl carbon. IRC calculations confirmed that this pathway leads to decarboxylation and formation of Products7 (Prod7).

The reaction yields CO_2_ and 4‐vinylguaiacol; however, in contrast to Mechanism B, the carboxyl‐derived fragment remains partially associated with the alumina surface. Consequently, the catalyst surface is not fully regenerated after the reaction, and residual surface species persist in the final state.

Comparison of the calculated Gibbs free‐energy profiles (Δ*G* ^‡^ = 336 kJ mol^−1^) indicates that Mechanism F is less favorable (Figure 9) than the acidic decarboxylation pathway (Mechanism B). Together with the incomplete regeneration of the catalytic surface, this suggests that the concerted acid–base route is unlikely to represent a major reaction channel under the studied conditions.

#### 2.7.3 Comparison of the Proposed Mechanisms

Overall, the DFT results provide a molecular‐level framework that is fully consistent with the experimentally observed catalytic behavior and deactivation trends reported in this work. The calculations demonstrate that among all investigated pathways (A–F), the acidic decarboxylation via Mechanism B, mediated by a coordinated water molecule within the aluminum coordination sphere, is the kinetically most favorable route for 4‐vinylguaiacol formation. This pathway exhibits the lowest activation free energy for the rate‐determining step, in agreement with TPD–MS measurements showing 4‐vinylguaiacol evolution at relatively low T_max_ values and with the experimentally derived activation energies reported for alumina.

In contrast, alternative reaction channels (Mechanisms C, D, and F) preferentially lead to strongly bound surface species rather than fully desorbed products. These surface‐bound intermediates provide a molecular basis for the experimentally observed decrease in catalytic activity after pyrolysis and regeneration, which was attributed to incomplete restoration of active surface sites and formation of persistent carbonaceous residues.

Importantly, Mechanism C offers a mechanistic explanation for the formation of surface‐anchored fragments through a “stitching” process, which can evolve into extended polyaromatic carbon domains. This finding is directly consistent with the experimental evidence presented in this study, including FTIR, XPS, TPD–MS, and TEM observations, which collectively indicate the development of a radially oriented polyaromatic carbon layer on alumina.

Taken together, the combined experimental and theoretical analysis confirms that alumina regeneration plays a critical role in controlling both catalytic efficiency and deactivation pathways in the pyrolysis of ferulic acid. Alumina acts as an active amphoteric surface that governs adsorption geometry, stabilizes key transition states, and directs the reaction network toward either selective formation of valuable aromatic products or accumulation of carbonaceous deposits. These insights provide a mechanistic basis for optimizing catalytic pyrolysis processes for lignin‐derived biomass conversion into high‐value chemicals.

#### 2.7.4 Relation of Calculated Reaction Energetics to Experimental Observations

The calculated activation barriers were further evaluated in relation to experimental observations obtained in the present work and to available literature data for ferulic acid decarboxylation on oxide surfaces and under non‐catalytic pyrolysis conditions.

For alumina, the evolution of 4‐vinylguaiacol was observed at *T*_max_ values of 145°C and 197°C for the initial catalyst, corresponding to apparent activation energies of 88 and 102 kJ mol^−1^, respectively, as estimated from TPD data (Table 2). For the regenerated catalyst, the corresponding desorption maxima were shifted to 193 and 242°C, yielding apparent activation energies of 96 and 107 kJ mol^−1^. The increase in both *T*_max_ and activation energy after regeneration is consistent with partial catalyst deactivation and a decrease in the concentration of the most active surface sites. Overall, these experimentally derived activation energies are in good agreement with the DFT results and support the proposed role of alumina in facilitating ferulic acid decarboxylation through stabilization of surface‐bound intermediates and transition states.

A comparison with other oxide catalysts further highlights the catalytic effect of alumina. For example, ferulic acid decarboxylation over nanoceria was reported to proceed through two distinct pathways with *T*_max_ values of approximately 120°C and 220°C, corresponding to activation energies of about 73 and 97 kJ mol^−1^, respectively [15]. In contrast, decarboxylation over silica occurs at substantially higher temperatures (*T*_max_≈408°C) and is characterized by an activation energy of approximately 133 kJ mol^−1^ and a pre‐exponential factor of 3.7 × 10^7^ s^−^^1^ [64]. The similar activation‐energy range observed for alumina and ceria indicates that both oxides effectively promote the decarboxylation process, whereas silica exhibits considerably lower catalytic activity.

An even more pronounced difference is observed when comparing catalytic conversion with the non‐catalytic pyrolysis of ferulic acid. According to the study [62], thermal decomposition of solid ferulic acid occurs over a broad temperature range of approximately 360°C–600°C, with a maximum decomposition rate near 450°C. Under these conditions, 4‐vinylguaiacol is not formed in significant amounts. Instead, the process proceeds predominantly through condensation, oligomerization, and subsequent decomposition of polymeric intermediates generated during heating. These observations indicate that direct thermal decarboxylation of ferulic acid is kinetically less favorable than the corresponding catalytic pathways occurring on oxide surfaces.

The relatively high thermal stability of ferulic acid is further evidenced by comparison with structurally related hydroxycinnamic acids. Under non‐catalytic pyrolysis conditions, caffeic acid and p‐coumaric acid readily undergo decarboxylation to form the corresponding vinyl phenols, exhibiting *T*_max_ values of approximately 160°C and 120°C and activation energies of 79 and 84 kJ mol^−1^, respectively [62]. In contrast, ferulic acid requires substantially higher temperatures and follows alternative decomposition routes involving oligomeric and polymeric intermediates, emphasizing the importance of catalytic surface interactions for efficient 4‐vinylguaiacol formation.

Overall, the combined experimental, computational, and literature data consistently demonstrate that oxide surfaces play a decisive role in promoting ferulic acid decarboxylation. The interaction of ferulic acid with surface active sites lowers the energetic requirements of the reaction, promotes selective decarboxylation at temperatures substantially lower than those required for non‐catalytic pyrolysis, where polymerization and carbonization pathways become dominant.

## 3 Conclusions

A combination of experimental and computational techniques was employed to investigate the catalytic pyrolysis of ferulic acid, a lignin‐derived model biomass compound, over alumina and the regeneration behavior of the catalyst surface. The catalytic performance of pristine and regenerated alumina was evaluated using TPD–MS, while mechanistic insights were obtained from DFT calculations.

Kinetic analysis revealed that the apparent activation energies for the formation of key pyrolysis products, including 4‐vinylguaiacol, guaiacol, phenol, benzene, toluene, cresol, and naphthalene, increase by 4–23 kJ mol^−^^1^ upon catalyst regeneration. This indicates a partial loss of accessible active sites and a modification of the surface reactivity after thermal treatment.

This decrease in activity is attributed to the formation and evolution of a carbonaceous overlayer on the alumina surface. The experimental evidence indicates that this layer is not static but undergoes a complex transformation pathway. Initially, ferulic acid phenolate complexes undergo a styrene‐like polymerization process, leading to the formation of a polymeric surface film. Upon further heating, this polymeric layer decomposes, resulting in the concurrent release of aromatic species detected by TPD–MS and the progressive development of extended polyaromatic carbon domains on the catalyst surface.

This mechanistic picture is strongly supported by complementary characterization techniques. TEM analysis reveals radially oriented, striped and filamentous features on alumina particles, consistent with the formation of non‐uniform carbonaceous structures. FTIR spectroscopy indicates an increasing contribution of aromatic C═C vibrations, while XPS analysis confirms the presence of extended π‐conjugation through the π–π* shake‐up feature in the C 1s region. Together, these results demonstrate the evolution of a structured polyaromatic carbon layer rather than random carbon deposition.

DFT calculations provide molecular‐level insight into the catalytic decarboxylation of ferulic acid to 4‐vinylguaiacol and rationalize the experimentally observed activation barriers. The calculations show that surface coordination of ferulic acid to aluminum centers plays a key role in stabilizing reactive intermediates and lowering the energetic barrier for decarboxylation. These results are consistent with the experimentally determined kinetics and confirm the importance of alumina surface chemistry in directing selective formation of aromatic products.

In addition, theoretical modeling suggests that strongly bound surface intermediates may contribute to catalyst deactivation through the formation of persistent surface‐bound species that evolve into carbonaceous deposits under reaction conditions. This is consistent with the experimentally observed incomplete regeneration of alumina and the accumulation of polyaromatic surface structures.

Overall, the combined experimental and theoretical results demonstrate that the catalytic pyrolysis of ferulic acid proceeds through a coupled sequence of surface reactions involving decarboxylation, polymerization, and carbon layer restructuring. The formation of polyaromatic domains plays a central role in catalyst deactivation, while alumina simultaneously governs both the selectivity of aromatic product formation and the evolution of the surface carbon phase. These findings provide a coherent molecular‐level understanding of reaction pathways and deactivation mechanisms in biomass‐derived aromatic conversion over oxide catalysts.

## 4 Experimental Section

In this work, nonporous fumed alumina (S_a_ = 111 m^2^g^−1^, d_a_∼40 nm, Degussa AG) was calcined at 450°C for two hours to remove various contaminants [29]. This alumina sample was used for ferulic acid (≥99%, Alfa Aesar) loading with a concentration of 0.6 mmol/g. Catalytic pyrolysis of ferulic acid was conducted from 20 to 500°C under TPD MS condition. This alumina sample was regenerated at 500°C for two hours in the air to oxidize surface coke. After the regeneration procedure, this alumina sample was again used to load ferulic acid (0.6 mmol/g) by mixing it with ferulic acid solutions in ethanol and then air drying. Its catalytic activity was investigated using a kinetic study by TPD MS from 20 to 750°C. These alumina samples (after pyrolysis and after regeneration) were studied by XPS, XRD, TEM, FTIR, and thermogravimetry.

TGA/TG/DTA analysis was conducted using a Q‐1500D analyzer (Hungary). Samples were heated from room temperature until 1000°C at a heating rate of 10°C/min in an air atmosphere. X‐ray diffractometry (XRD) evaluates the crystalline structure, nature of the phase, lattice parameters and crystalline grain size. All samples were dried and fine‐grained before the measurement. The diffraction patterns were obtained utilizing a PANalytical X'Pert3 (Malvern) diffractometer, which was equipped with a CoKα radiation source (with a wavelength of 1.79 Å) and operated at 40 mA. The particles composition is determined by comparing the reference patterns (from International Centre for Diffraction Data database) with the position and intensity of the peaks. Transmission Electron Microscopy (TEM) was used to observe the structure and morphology of particles. After redispersion in deionized water, samples suspensions were deposited onto carbon‐coated copper grids via drop‐casting and subsequently air‐dried at room temperature. TEM images were captured using a JEOL 1200 EX microscope with a 120 kV acceleration voltage. Size distributions from TEM images were measured using the image analysis software ImageJ. For each sample, measurements of at least 200 particles were taken manually to obtain the average diameter (DTEM) and its standard deviation (σDTEM). FT‐IR spectroscopy study was conducted on a Thermo Nicolet Nexus Fourier‐transform IR spectrometer in diffuse reflection mode with the number of scans set at 50, a resolution of ±4 cm^−1^, the range 4000–400 cm^−1^ and the scan velocity of 0.5 cm/s. Samples of alumina were mixed with KBr (pre‐calcined at 500°C for 2 h) at a ratio of 1:10. Ferulic acid was mixed with KBr at a ratio of 1:100.

X‐ray photoelectron spectra (XPS) were collected using a Kratos Axis Ultra DLD system with a monochromatic AlKα X‐ray source operating at 120 W. Data was collected in the hybrid mode of operation, using a combination of magnetic and electrostatic lenses, at pass energies of 40 and 160 eV for high‐resolution and survey spectra, respectively. Magnetically confined charge compensation was used to minimize sample charging and the resulting spectra were calibrated to the Al 2p line for Al_2_O_3_ at 74.6 eV.

TPD–MS experiments were carried out using an MX‐7304A mass spectrometer (Sumy, Ukraine) equipped with an electron ionization source and adapted for investigations of catalytic and direct pyrolysis processes. The kinetic parameters, including the temperature of maximum desorption rate (*T*_max_), reaction order (*n*), activation energy (*E^≠^*), pre‐exponential factor (*ν*_0_), and activation entropy change (Δ*S^≠^*), were evaluated from TPD–MS data using the Arrhenius plot approach as previously described in earlier studies including our own works [30, 31, 32, 33, 34, 35].

Approximately 20 mg of sample was placed in a quartz molybdenum ampoule, which was evacuated at room temperature to a pressure of ∼5 × 10^−^^5^ Pa. The sample was then subjected to linear programmed heating from 20 to ∼750°C at a rate of 0.17°C s^−^^1^. Volatile products generated during pyrolysis entered the mass spectrometer's ionization chamber through a high‐vacuum valve, where they underwent electron‐impact ionization and fragmentation. The resulting ion currents were detected using a VEU‐6 secondary electron multiplier, while mass spectra were continuously recorded and processed using a computerized data acquisition system over the mass range of 1–210 Da, yielding up to 240 spectra per experiment. The combination of slow heating and efficient removal of volatile species ensured that diffusion limitations were negligible, allowing the ion current intensity to be considered proportional to the desorption rate.

Density functional theory (DFT) calculations were performed using Gaussian 09 software. The wB97XD functional with Grimme's D2 dispersion correction [67, 68] was employed in combination with the 6–311++G(d,p) basis set. All molecular systems were fully optimized without geometric constraints.

The alumina surface was modeled using an Al_2_(OH)_6_ cluster, which represents a minimal but chemically meaningful model of surface aluminum and hydroxyl groups, consistent with previous studies [41]. Transition states (TS) were located using the Berny algorithm and the GEDIIS procedure in redundant internal coordinates [69, 70]. Initial TS guesses were generated by chemically motivated distortions of the corresponding prereaction complexes, focusing on atoms directly involved in bond‐breaking and bond‐forming events (carboxyl group, aluminum center, and surface hydroxyl species). No geometric constraints were imposed during optimization.

All stationary points were verified by vibrational frequency analysis. Minima were confirmed by the absence of imaginary frequencies, while transition states were identified by a single imaginary frequency corresponding to the reaction coordinate. The nature of all transition states was further validated using intrinsic reaction coordinate (IRC) calculations [71].

Thermodynamic corrections were obtained from harmonic frequency calculations. Gibbs free energies were evaluated at 421 K for all reaction pathways. Activation free energies (Δ*G* ^‡^) were defined as the difference between the Gibbs free energy of the prereaction complex (or intermediate for multistep processes) and the corresponding transition state. Reaction free energies (ΔG) were calculated from the differences between reactants, intermediates, and products along each step of the reaction pathway.

Bond order analysis was performed using natural bond orbital (NBO) calculations (NBO 3.1) [72]. Molecular geometries and vibrational modes were visualized using MaSK version 1.3.0 [73], with special attention to bonds undergoing formation or cleavage during decarboxylation and surface‐assisted transformations.

This computational protocol was applied consistently to gas‐phase decarboxylation and alumina‐assisted surface mechanisms to ensure direct comparability across all pathways.

## Author Contributions

**Tetiana V. Kulik**: writing – original draft, writing – review and editing, visualization, project administration, supervision, methodology, investigation, formal analysis, conceptualization, funding acquisition. **Borys B. Palianytsia**: methodology, investigation, visualization, formal analysis, writing – review. **Mykola M. Ilchenko**: methodology, investigation, formal analysis, writing – review and editing. **Nataliia S. Nastasiienko**: investigation, formal analysis, writing – review. **David J. Morgan**: methodology, investigation, formal analysis, writing – review. **Liudmila P. Storozhuk**: methodology, investigation, formal analysis, writing – review. **David J. Willock**: writing – review and editing, conceptualization. **Greg Shaw**: investigation, formal analysis. **Duncan F. Wass**: writing – review and editing, conceptualization. **Rafael Luque**: writing – review and editing, formal analysis, conceptualization. **Philip R. Davies**: writing – review and editing, methodology, formal analysis, project administration, conceptualization. **Mykola T. Kartel**: writing – review and editing.

## Conflicts of Interest

The authors declare no conflicts of interest.

## Supporting information




**Supporting File 1**:chem71397‐sup‐0001‐SuppMat.pdf
XRD spectra, XPS spectra with Tables S1,S2 of relative carbon and oxygen concentrations in each sample and photos for all samples (Figures S1–S3), DFT calculations with Figures S4 and the Cartesian coordinates of all optimized structures.

## Data Availability

The data that support the findings of this study are available from the corresponding author upon reasonable request.
