# Supplementary material for: Mechanistic Insights Into Sustainable Catalytic Pyrolysis of Biomass‐Derived Ferulic Acid: The Role of Alumina Regeneration
Source: Chemistry. 2026 Jul 14;32(39):e71397. doi: 10.1002/chem.71397 (PMC13715462; doi:10.1002/chem.71397)
Supplement: Supplementary file 1 [file CHEM-32-e71397-s001.pdf]

Supporting Information

Mechanistic Insights into Sustainable Catalytic Pyrolysis of Biomass-Derived Ferulic Acid: The Role of Alumina Regeneration

Tetiana V. Kulik*[a,b], Borys B. Palianytsia[b], Mykola M. Ilchenko[b,c], Nataliia S. Nastasiienko[b], David J. Morgan[a], Liudmila P. Storozhuk[d,e], David J. Willock[a], Greg Shaw[a], Duncan F. Wass[a], Rafael Luque[f,g], Philip R. Davies[a], Mykola T. Kartel[b]

[a]Cardiff Catalysis Institute, School of Chemistry, Cardiff University, Main Building, Park Place, Cardiff, CF10 3AT, UK

[b]Chuiko Institute of Surface Chemistry, National Academy of Science of Ukraine, Kyiv 03164, Ukraine.

[c]Institute of Molecular Biology and Genetics, National Academy of Science of Ukraine, 150 Zabolotnogo Str., Kyiv 03680, Ukraine

[d]Biophysics Group, Department of Physics and Astronomy, University College London, London, UK

[e]UCL Healthcare Biomagnetics and Nanomaterials Laboratories, London, W1S 4BS, UK

[f]National University of Science and Technology Polytehnica Bucharest, 1-7 Gh. Polizu street, Bucharest, Romania

[g]Universidad ECOTEC, Km. 13.5 Samborondón, Samborondón, EC092302, Ecuador

*****Correspondence: KulykT@cardiff.ac.uk (T.K.)

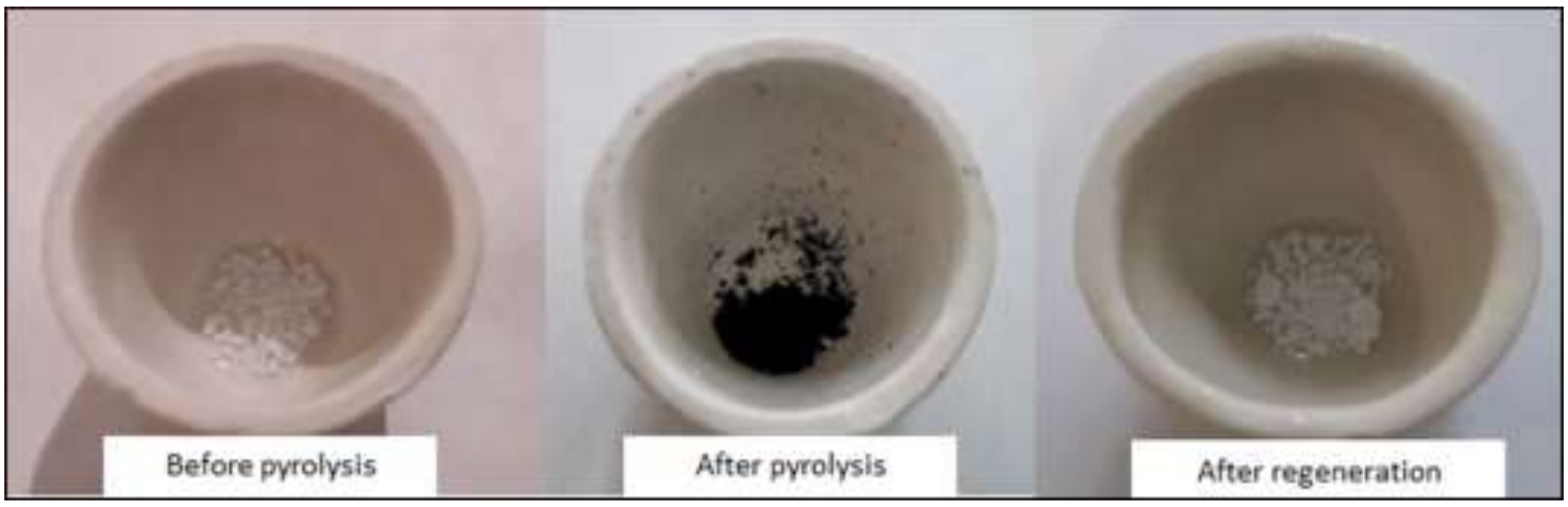

Figure S1. The alumina samples before and after pyrolysis, and after regeneration.

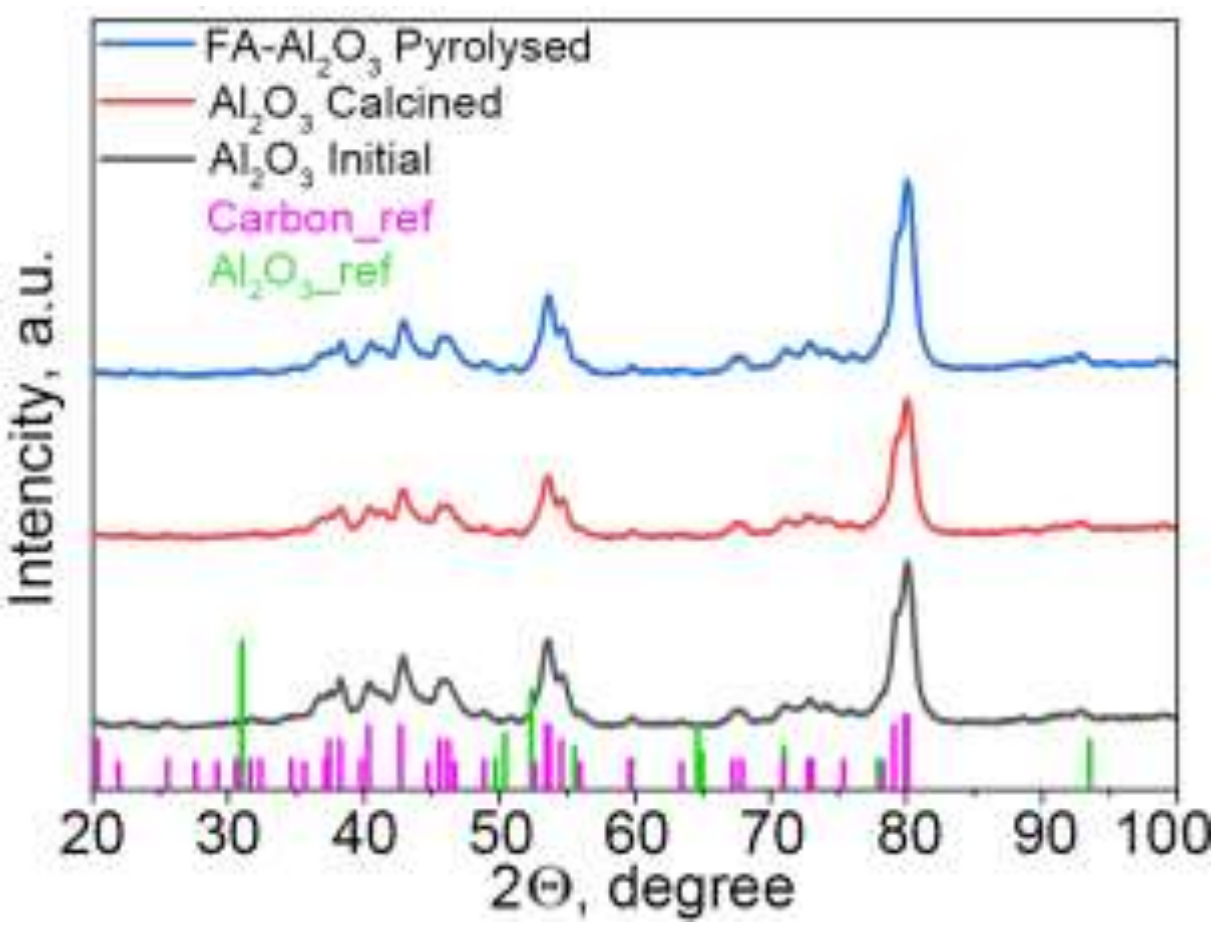

Figure S2. X-Ray diffraction analysis (XRD).

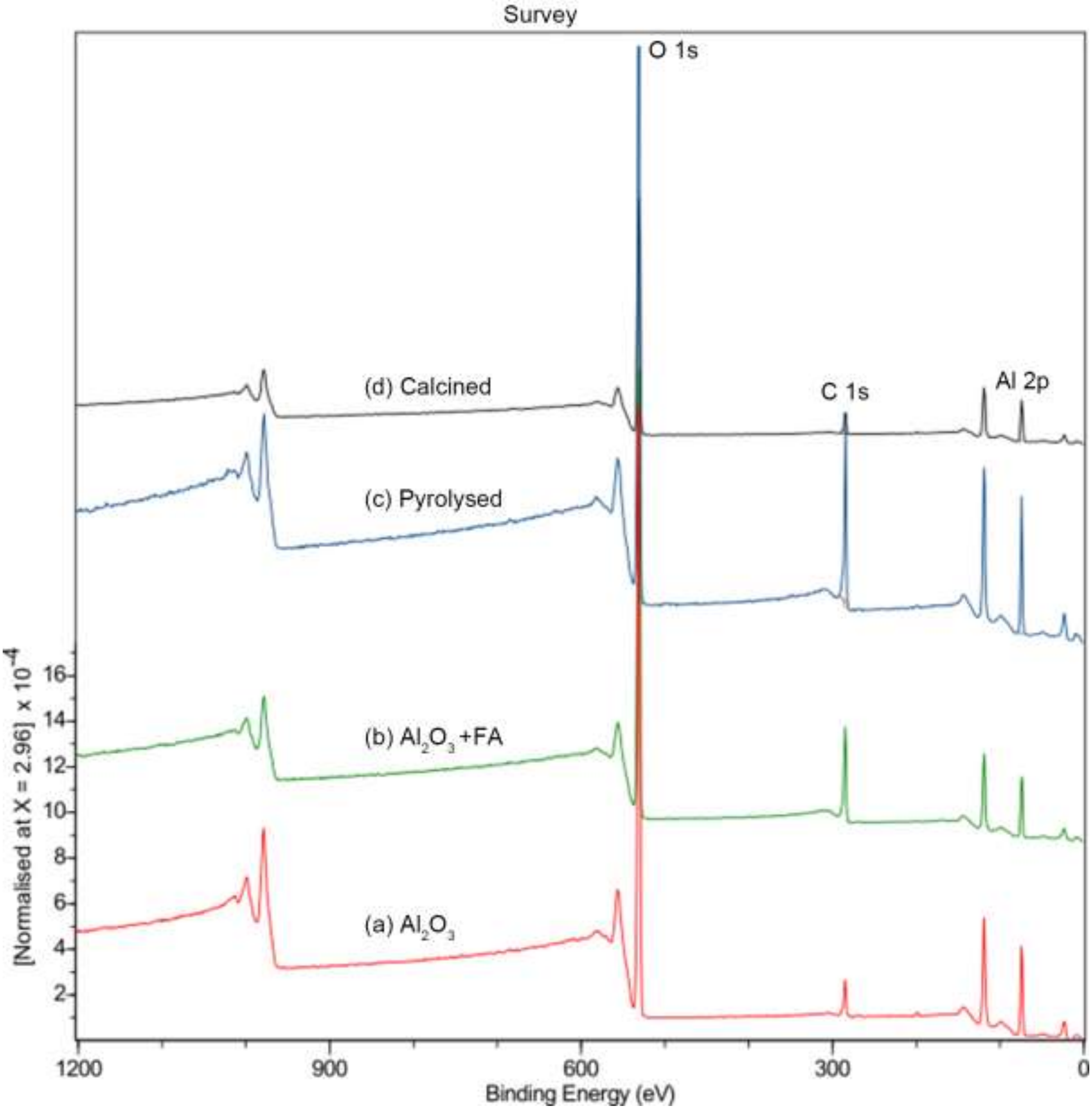

Figure S3. XPS data for samples: initial alumina (a); Fer/Al before (b) and after (c) pyrolysis; regenerated alumina (d).

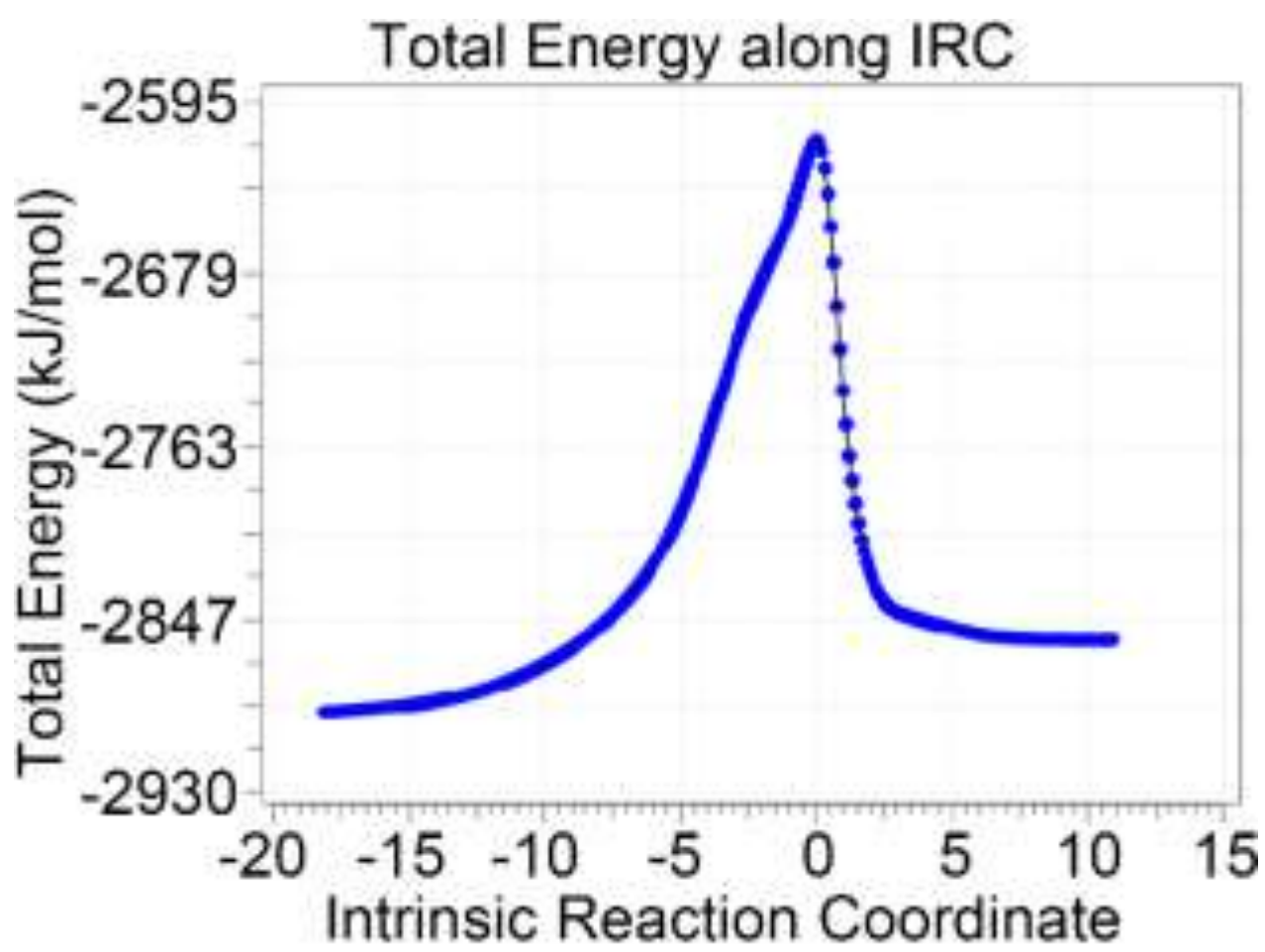

Figure S4. Total electron energy values along the internal coordinate of the reaction for the mechanism of ferulic acid decarboxylation in the gas phase.

Table S1. XPS deconvolution peaks C 1s (% of total peak area). Relative carbon concentrations in each sample.

Relative carbon concentrations in each sample			
Sample Identifier	Peak	Position Binding Energy (eV)	%At. Conc.
(a) Al2O3			
C:	C-C, CHx	285.0	67
C:	C-OH	286.3	33
C:	C-O/C=O	289.1	<1
(b) Al2O3 + FA			
C:	C 1s (aromatic)	284.6	43
C:	C 1s (aromatic)	291.4	0
C:	sp^2, C=C, C-C, CHx	285.1	35
C:	C-OH	286.4	18
C:	CO2H, COO^-	288.5	4
C:	CO3	289.5	0
(c) Pyrolysed			
C:	C 1s (aromatic)	284.5	50
C:	C 1s (aromatic)	291.4	3
C:	C=C, C-C, CHx	285.0	35
C:	C-OH	286.7	6
C:	CO2, C=O	288.4	3
C:	CO3	289.4	3
(d) Calcined			
C:	C-C, CHx	284.9	71
C:	C-OH	286.2	22
C:	CO3	289.1	7

Table S2. XPS deconvolution peaks O 1s (% of total peak area). Relative oxygen concentrations in each sample.

Sample Identifier	Peak	Position Binding Energy (eV)	%At. Conc.
(a) Al2O3			
	O2-	531.2	75
	OH	532.5	25
(b) Al2O3 + FA			
	O2-	531.1	46
	O 1s	532.0	42
	C-O, CO2H, COO^-	533.5	12
(c) Pyrolysed			
	O2-	531.2	61
	O 1s	532.2	34
	C=O	533.7	5
(d) Calcined			
	O2-	531.2	76
	OH	532.6	24

Cartesian coordinates (Å) of all optimized prereaction complexes, intermediates, transition states, and products discussed in Section 2.5 and shown in Figures 8 and 9.

Prereaction1

O	4.531122	1.292065	-0.409301
H	2.003384	1.395819	0.016274
C	3.883517	0.356405	-0.030673
O	4.504213	-0.782338	0.355541
C	2.401483	0.387917	0.026132
C	1.606580	-0.687559	-0.014975
C	0.143432	-0.712956	-0.023823
C	-0.628679	0.460475	0.025780
C	-2.005644	0.385418	0.017838
C	-2.652258	-0.864746	-0.039755
C	-1.897723	-2.021308	-0.089390
C	-0.509510	-1.941776	-0.081851
O	-2.864301	1.440125	0.061198
O	-3.998548	-0.927755	-0.046813
H	3.880476	-1.374982	0.781707
H	2.060341	-1.676904	-0.072832
H	-0.142672	1.426060	0.069457
H	-2.407428	-2.975704	-0.134839
H	0.072402	-2.856255	-0.123430
H	-4.340396	-0.027817	-0.010015
C	-2.323830	2.746147	0.112501
H	-1.719497	2.885318	1.014852
H	-1.716947	2.954556	-0.774617
H	-3.173745	3.425022	0.137738

TS1

O	-4.336997	0.800715	1.156980
H	-2.010024	1.404134	-0.450280
C	-3.959857	0.292513	0.137358
O	-4.530327	-0.363310	-0.793320
C	-2.424306	0.406523	-0.337604
C	-1.601587	-0.672108	-0.104813
C	-0.167242	-0.707093	-0.042025
C	0.614386	0.467758	-0.083510
C	1.984591	0.382690	-0.022121
C	2.615406	-0.878516	0.081457
C	1.853901	-2.035779	0.126671
C	0.473160	-1.947237	0.067711
O	2.858953	1.420132	-0.044506
O	3.950457	-0.949889	0.141966
H	-3.152636	-0.069549	-1.249448
H	-2.097314	-1.635785	0.014110
H	0.130088	1.432581	-0.151803
H	2.358970	-2.989577	0.211007
H	-0.122792	-2.852254	0.104920
H	4.305759	-0.053871	0.104194
C	2.340211	2.736131	-0.127516

H	1.774639	2.874115	-1.054418
H	1.703463	2.958988	0.734287
H	3.201184	3.400746	-0.125133

Products1

O	1.840837	1.945649	-1.072416
H	2.506214	0.956700	1.718392
C	1.973697	0.937072	-1.627133
O	2.113371	-0.056363	-2.199228
C	2.851078	-0.072209	1.704293
C	2.092071	-1.081582	1.277952
C	0.713251	-1.006523	0.769460
C	-0.052511	0.166715	0.860143
C	-1.324485	0.214108	0.324122
C	-1.876493	-0.916969	-0.301886
C	-1.134469	-2.080105	-0.378866
C	0.150104	-2.120834	0.153858
O	-2.155414	1.295168	0.339013
O	-3.124561	-0.864126	-0.819971
H	3.860905	-0.249349	2.054517
H	2.521337	-2.081202	1.275841
H	0.359219	1.039494	1.349214
H	-1.570431	-2.945478	-0.862984
H	0.727740	-3.035352	0.075074
H	-3.469946	0.021904	-0.668846
C	-1.667486	2.504118	0.886411
H	-1.443811	2.390615	1.952449
H	-0.770799	2.840879	0.356104
H	-2.462843	3.236415	0.760932

Prereaction2

Al	-1.891442	-2.087051	-0.843853
O	-2.825608	-1.768183	0.679182
O	-2.006869	-0.249919	-0.942576
O	-0.273851	-2.634421	-0.532898
H	-1.956428	0.300231	-1.720063
H	-2.124852	1.041299	2.718349
H	-2.698330	-2.186790	1.528084
O	-2.657435	-2.912404	-2.143726
H	-3.586924	-2.862921	-2.347234
Al	-2.946474	0.154376	0.650668
O	-4.645166	0.406458	0.474068
H	-5.007893	1.278500	0.346727
O	-1.993332	0.312738	2.117033
H	0.090603	-3.314220	-1.095983
O	-2.525430	2.200202	0.111840
H	-0.296516	1.164336	0.858616
C	-1.430075	2.728355	-0.037436
O	-1.412309	3.996954	-0.443927
C	-0.178985	2.012111	0.193828
C	0.976706	2.263718	-0.441836
C	2.179736	1.450910	-0.343835
C	2.154585	0.182898	0.266214
C	3.311912	-0.557569	0.361086
C	4.522374	-0.052814	-0.159173
C	4.547396	1.186346	-0.774798

C	3.378430	1.930675	-0.870705
O	3.427293	-1.786568	0.916998
O	5.644481	-0.788180	-0.065046
H	-0.517381	4.346230	-0.402281
H	1.053190	3.111403	-1.124242
H	1.222978	-0.235666	0.623485
H	5.484983	1.552191	-1.174379
H	3.404508	2.901464	-1.354432
H	5.421663	-1.608549	0.390573
C	2.288111	-2.332741	1.578716
H	1.444576	-2.449483	0.891393
H	2.604034	-3.304495	1.953115
H	1.999359	-1.693502	2.419736

TS2

Al	-0.992568	-2.134327	-0.831259
O	-2.132231	-2.089011	0.565506
O	-1.572327	-0.388726	-1.003212
O	0.657779	-2.265349	-0.299942
H	-1.742328	0.054730	-1.831862
H	-2.594497	0.700100	2.624202
H	-2.018060	-2.462862	1.436114
O	-1.339585	-3.094813	-2.216670
H	-2.216329	-3.308201	-2.522947
Al	-2.809208	-0.267858	0.453313
O	-4.457476	-0.622197	0.057673
H	-5.062735	0.089432	-0.134260
O	-2.125558	0.108432	2.040000
H	1.260154	-2.798872	-0.813980
O	-2.985028	1.710880	-0.051539
H	-0.487691	1.729838	1.389783
C	-2.080675	2.547509	0.064731
O	-2.143053	3.808035	-0.067096
C	-0.643328	2.235163	0.442605
C	0.309310	2.163218	-0.560131
C	1.589089	1.550550	-0.457790
C	1.846337	0.585866	0.541677
C	3.070280	-0.031677	0.591718
C	4.074600	0.316889	-0.340354
C	3.826322	1.260776	-1.331229
C	2.583941	1.858607	-1.400327
O	3.447199	-1.001829	1.452600
O	5.260642	-0.293331	-0.284089
H	-0.820505	3.552651	0.393034
H	0.077863	2.653959	-1.505915
H	1.054606	0.280142	1.212064
H	4.610892	1.490157	-2.040871
H	2.376936	2.583410	-2.179962
H	5.235091	-0.931808	0.440319
C	2.454948	-1.541543	2.325802
H	1.618564	-1.930835	1.738531
H	2.947928	-2.344122	2.870158
H	2.115354	-0.778186	3.032835

Intermediate2

Al	-0.456740	2.241122	-0.846832

O	0.422882	2.450540	0.727280
O	0.610901	0.762339	-0.970612
O	-2.128204	1.766490	-0.698714
H	0.837892	0.238600	-1.735623
H	1.245802	0.782357	2.719434
H	0.857570	3.280715	0.926625
O	-0.237027	3.474965	-2.030157
H	0.606309	3.865498	-2.242790
Al	1.850072	1.002869	0.458185
O	3.012352	2.295152	0.283844
H	3.915193	2.082065	0.060650
O	1.467301	0.201228	1.995058
H	-2.759589	2.285300	-1.193544
O	2.853009	-0.420257	-0.277376
H	1.762551	-1.864358	1.514172
C	2.949073	-1.684949	-0.353810
O	3.636189	-2.342858	-1.098479
C	1.996488	-2.494476	0.658461
C	0.864287	-2.866189	-0.170652
C	-0.411903	-2.334489	-0.165310
C	-0.861639	-1.413056	0.834041
C	-2.111130	-0.879488	0.741058
C	-2.928821	-1.181353	-0.386907
C	-2.523727	-2.116613	-1.351116
C	-1.289801	-2.691381	-1.232544
O	-2.714870	-0.050316	1.609047
O	-4.086746	-0.578984	-0.508067
H	2.584579	-3.372508	0.927339
H	1.084675	-3.592046	-0.952852
H	-0.181968	-1.101715	1.620370
H	-3.189056	-2.334704	-2.176166
H	-0.949766	-3.406390	-1.973613
H	-4.116977	0.123922	0.161915
C	-1.904767	0.645244	2.553169
H	-1.139770	1.230614	2.038982
H	-2.582509	1.303269	3.092214
H	-1.442923	-0.058404	3.252339
TS3			
Al	2.689548	1.867337	-0.165228
O	3.874943	0.591391	0.378087
O	1.754282	0.382145	-0.705641
O	1.723688	2.569885	1.090843
H	0.898331	0.329385	-1.124089
H	3.047543	-1.083268	2.282058
H	4.799965	0.577566	0.137084
O	3.283482	2.973117	-1.340387
H	3.798688	2.748211	-2.109236
Al	2.853451	-1.018423	-0.047852
O	4.049564	-1.657401	-1.125184
H	3.943757	-2.493234	-1.570295
O	2.516098	-1.494424	1.603816
H	1.828709	3.478580	1.360651
O	1.353962	-2.269556	-0.672600
H	0.522792	-2.599268	1.831020

C	0.156850	-2.431395	-0.533580
O	-0.822573	-2.735399	-1.124043
C	-0.360197	-2.203519	1.338896
C	-0.452974	-0.827960	1.289633
C	-1.553796	-0.023659	0.849953
C	-2.784978	-0.585597	0.446345
C	-3.783627	0.230447	-0.025478
C	-3.586181	1.628932	-0.093111
C	-2.379751	2.185012	0.305231
C	-1.364770	1.364603	0.769932
O	-5.007359	-0.164518	-0.464482
O	-4.570255	2.414065	-0.550633
H	-1.251937	-2.818368	1.316284
H	0.456290	-0.287561	1.540769
H	-2.925263	-1.657168	0.485634
H	-2.250885	3.257744	0.234188
H	-0.407399	1.794834	1.058349
H	-5.317757	1.853971	-0.791252
C	-5.281233	-1.553773	-0.514353
H	-4.563170	-2.069123	-1.159780
H	-5.259097	-1.991386	0.488881
H	-6.282272	-1.649786	-0.929252

Products2

Al	-3.330205	0.820081	0.195430
O	-3.569242	-0.811298	-0.668983
O	-1.626615	0.138674	0.411419
O	-3.233027	2.171885	-0.866676
H	-0.865864	0.714106	0.189324
H	-0.698348	-2.322705	-2.288213
H	-4.374877	-1.324123	-0.694080
O	-4.341298	0.984666	1.568979
H	-4.411009	0.383721	2.304313
Al	-1.874149	-1.509504	-0.368106
O	-2.189140	-2.883476	0.612002
H	-1.510038	-3.440554	0.981967
O	-0.799869	-1.560772	-1.723906
H	-3.785820	2.942532	-0.769423
O	0.684718	-2.016432	1.457478
H	1.827965	-2.784857	-1.194141
C	1.201535	-1.191079	2.086951
O	1.706041	-0.377558	2.731384
C	2.908844	-2.880893	-1.164047
C	3.701964	-1.859230	-0.845052
C	3.269648	-0.492830	-0.507635
C	1.971575	-0.040995	-0.768972
C	1.597370	1.233753	-0.394392
C	2.487456	2.100779	0.244727
C	3.782250	1.666312	0.490985
C	4.161850	0.386380	0.112526
O	0.309922	1.712059	-0.578222
O	2.097973	3.340019	0.613703
H	3.324717	-3.854338	-1.395608
H	4.776251	-2.026721	-0.803121
H	1.235812	-0.674494	-1.255504

H	4.475913	2.336626	0.983531
H	5.175144	0.056187	0.316699
H	1.163107	3.426367	0.395507
C	-0.125813	1.814806	-1.950290
H	-1.130468	2.234821	-1.909765
H	-0.157914	0.822224	-2.404990
H	0.556253	2.469540	-2.497965

Prereaction3

Al	-4.794186	-1.220315	-0.434322
O	-4.940300	0.438126	0.260826
O	-3.198311	-0.515520	-1.015222
O	-4.296566	-2.167754	0.960434
H	-2.400566	-0.954235	-1.306232
H	-3.422584	-0.944608	1.805793
H	-5.602747	1.122095	0.315524
O	-5.929077	-1.922627	-1.491910
H	-6.276709	-1.587741	-2.311962
Al	-3.071015	0.956051	0.184622
O	-3.476147	2.625892	0.084963
H	-2.716536	3.209430	-0.005552
O	-2.914834	-0.092115	1.844211
H	-4.734436	-2.952248	1.279357
O	-1.232812	0.869696	0.134077
H	-1.988002	-0.292312	2.007542
C	-0.344936	1.826280	-0.034027
O	-0.622221	3.006580	-0.164533
C	1.072276	1.386665	-0.055303
C	1.453388	0.111018	0.058835
C	2.819069	-0.417789	0.047787
C	3.945489	0.410299	-0.096010
C	5.213319	-0.133697	-0.100048
C	5.395078	-1.522453	0.039574
C	4.292075	-2.342294	0.181118
C	3.015204	-1.789930	0.184552
O	6.379587	0.558392	-0.231489
O	6.639981	-2.045419	0.034499
H	1.778436	2.200402	-0.176417
H	0.666983	-0.631423	0.171964
H	3.816136	1.479053	-0.204447
H	4.445350	-3.409250	0.287467
H	2.156005	-2.442710	0.295783
H	7.265665	-1.321242	-0.073683
C	6.312717	1.962816	-0.378696
H	5.757910	2.237136	-1.281938
H	5.845203	2.427609	0.495559
H	7.341046	2.307986	-0.466181

TS4

Al	-2.875769	-1.962586	-0.152011
O	-3.400123	-0.855425	1.195626
O	-2.506234	-0.373010	-0.970270
O	-1.320355	-2.706241	0.092719
H	-2.329083	-0.120897	-1.873490
H	-1.696899	0.546111	2.448963
H	-4.277629	-0.866146	1.576137

O	-4.076689	-3.014188	-0.791916
H	-4.977112	-2.780273	-0.995919
Al	-2.909982	0.909635	0.392275
O	-4.547182	1.479443	0.451841
H	-4.763723	2.354911	0.141697
O	-1.702118	1.158328	1.714292
H	-1.260221	-3.650805	0.215483
O	-2.027069	2.186687	-0.669748
H	-0.437976	1.643560	1.086128
C	-0.857792	2.716752	-0.675185
O	-0.511231	3.726167	-1.240616
C	0.252526	1.966987	0.131764
C	0.544718	0.650112	-0.244204
C	1.778013	-0.041543	-0.181957
C	3.015476	0.623646	0.003389
C	4.171135	-0.108595	0.071025
C	4.122407	-1.523440	-0.027508
C	2.911070	-2.179713	-0.200923
C	1.743777	-1.444856	-0.287643
O	5.429813	0.372337	0.223729
O	5.255185	-2.222829	0.042820
H	1.081106	2.618646	0.394877
H	-0.305625	0.034536	-0.534171
H	3.040096	1.703699	0.062693
H	2.908990	-3.260252	-0.266059
H	0.781816	-1.944057	-0.391741
H	5.990065	-1.607569	0.158581
C	5.603633	1.776667	0.317385
H	5.246501	2.273524	-0.589836
H	5.079744	2.175284	1.191646
H	6.672960	1.941840	0.426898

Intermediate3

Al	2.489845	1.864472	-0.281232
O	3.779997	0.697627	0.220818
O	1.574653	0.342624	-0.721345
O	1.586253	2.549878	1.043833
H	0.878848	0.188750	-1.355800
H	3.176906	-0.762957	2.202721
H	4.646945	0.656134	-0.181942
O	2.884515	2.989444	-1.524983
H	3.412172	2.801164	-2.295306
Al	2.766995	-1.026196	-0.077664
O	4.024512	-1.575721	-1.149517
H	3.932233	-2.410452	-1.601461
O	2.580653	-1.273230	1.658393
H	1.781932	3.428769	1.359709
O	1.401892	-2.286745	-0.554789
H	0.695077	-2.401932	1.930185
C	0.192321	-2.440630	-0.221562
O	-0.745373	-2.800791	-0.900209
C	-0.161353	-2.106688	1.326392
C	-0.343250	-0.671508	1.383042
C	-1.465868	0.038039	0.965095
C	-2.620429	-0.617124	0.444779

C	-3.648506	0.130122	-0.050297
C	-3.574768	1.551009	-0.014787
C	-2.450279	2.201875	0.492122
C	-1.398078	1.455229	0.968556
O	-4.791005	-0.328317	-0.612688
O	-4.588370	2.259533	-0.490281
H	-1.064417	-2.676930	1.532355
H	0.522795	-0.095136	1.700537
H	-2.641462	-1.697164	0.394366
H	-2.421341	3.283663	0.471730
H	-0.478119	1.936413	1.297315
H	-5.261253	1.649988	-0.823361
C	-4.913297	-1.731836	-0.812289
H	-4.081190	-2.109744	-1.413069
H	-4.946589	-2.254707	0.148141
H	-5.852227	-1.878793	-1.340617

TS5

Al	2.689548	1.867337	-0.165228
O	3.874943	0.591391	0.378087
O	1.754282	0.382145	-0.705641
O	1.723688	2.569885	1.090843
H	0.898331	0.329385	-1.124089
H	3.047543	-1.083268	2.282058
H	4.799965	0.577566	0.137084
O	3.283482	2.973117	-1.340387
H	3.798688	2.748211	-2.109236
Al	2.853451	-1.018423	-0.047852
O	4.049564	-1.657401	-1.125184
H	3.943757	-2.493234	-1.570295
O	2.516098	-1.494424	1.603816
H	1.828709	3.478580	1.360651
O	1.353962	-2.269556	-0.672600
H	0.522792	-2.599268	1.831020
C	0.156850	-2.431395	-0.533580
O	-0.822573	-2.735399	-1.124043
C	-0.360197	-2.203519	1.338896
C	-0.452974	-0.827960	1.289633
C	-1.553796	-0.023659	0.849953
C	-2.784978	-0.585597	0.446345
C	-3.783627	0.230447	-0.025478
C	-3.586181	1.628932	-0.093111
C	-2.379751	2.185012	0.305231
C	-1.364770	1.364603	0.769932
O	-5.007359	-0.164518	-0.464482
O	-4.570255	2.414065	-0.550633
H	-1.251937	-2.818368	1.316284
H	0.456290	-0.287561	1.540769
H	-2.925263	-1.657168	0.485634
H	-2.250885	3.257744	0.234188
H	-0.407399	1.794834	1.058349
H	-5.317757	1.853971	-0.791252
C	-5.281233	-1.553773	-0.514353
H	-4.563170	-2.069123	-1.159780
H	-5.259097	-1.991386	0.488881

H	-6.282272	-1.649786	-0.929252

Products3

Al	3.195990	1.033497	-0.223082
O	3.470535	-0.597860	0.624615
O	1.744308	0.101484	-0.862519
O	2.658944	2.293393	0.825027
H	0.872817	0.447447	-1.071242
H	0.504643	-1.460677	1.735297
H	4.308821	-0.974262	0.884463
O	4.456360	1.447515	-1.307768
H	4.785185	0.912848	-2.024215
Al	2.030966	-1.551727	-0.077197
O	2.691003	-2.738904	-1.123569
H	2.312212	-3.596324	-1.290527
O	0.787732	-2.023825	1.013379
H	3.131540	3.117634	0.909225
O	-0.724823	-1.616040	-1.510489
H	0.442979	0.157119	4.144623
C	-1.402003	-2.168255	-0.748843
O	-2.113489	-2.731267	-0.033605
C	-0.301016	0.210798	3.358606
C	-0.085828	0.956849	2.272846
C	-0.988399	1.129915	1.122504
C	-2.210126	0.448949	1.008380
C	-3.010728	0.631752	-0.103151
C	-2.608670	1.494243	-1.138766
C	-1.409698	2.174071	-1.028774
C	-0.609692	1.996818	0.098174
O	-4.210034	0.025934	-0.329549
O	-3.391743	1.657988	-2.226803
H	-1.214551	-0.354169	3.517699
H	0.852912	1.501489	2.189017
H	-2.524500	-0.232534	1.788210
H	-1.119331	2.850246	-1.824263
H	0.326223	2.538347	0.192349
H	-4.182074	1.120716	-2.106773
C	-4.793757	-0.723429	0.721831
H	-4.187311	-1.600824	0.962325
H	-4.923738	-0.101936	1.613864
H	-5.767089	-1.045754	0.356964

Prereaction4

Al	5.017942	-0.743771	0.108813
O	3.400665	-1.580083	0.269118
O	3.895560	0.670374	-0.245786
O	5.790894	-0.653152	1.645630
H	4.053980	1.585148	-0.465916
H	1.955139	-0.118363	2.446913
H	3.216125	-2.274379	0.897461
O	6.041157	-1.176680	-1.206810
H	5.763021	-1.273747	-2.112293
Al	2.191711	-0.040788	0.057611
O	1.220072	-0.652085	-1.271890
H	1.426384	-1.524596	-1.597529
O	1.464182	0.007477	1.640366

H	6.731323	-0.755587	1.759299
O	1.584017	1.865131	-0.353874
H	-0.537121	0.478333	-0.715651
C	0.453399	2.332642	-0.403943
O	0.353960	3.657684	-0.462119
C	-0.744493	1.499197	-0.413021
C	-1.952903	1.912845	-0.006273
C	-3.151840	1.088441	0.079512
C	-3.104381	-0.300658	-0.134076
C	-4.257928	-1.049290	-0.055501
C	-5.491010	-0.431589	0.236175
C	-5.540333	0.933232	0.453987
C	-4.374279	1.685681	0.380405
O	-4.349520	-2.394165	-0.230246
O	-6.612060	-1.173060	0.311401
H	-0.556428	3.916050	-0.630471
H	-2.093388	2.941228	0.328789
H	-2.157624	-0.781767	-0.341229
H	-6.494302	1.390831	0.684450
H	-4.421267	2.754940	0.557315
H	-6.374860	-2.091594	0.141765
C	-3.160591	-3.115828	-0.499745
H	-2.703354	-2.780551	-1.436033
H	-2.443054	-3.007121	0.319490
H	-3.455339	-4.159128	-0.591314
TS6			
Al	4.080634	-1.249058	0.448890
O	2.269186	-1.486141	0.519753
O	3.525505	0.406595	-0.140958
O	4.864730	-1.130330	1.973298
H	3.975878	1.248783	-0.163080
H	0.620983	0.036139	1.993808
H	1.808901	-2.275967	0.246526
O	4.874994	-2.242481	-0.717028
H	4.634306	-2.325886	-1.634296
Al	1.660986	0.270197	-0.109835
O	0.757383	-0.324941	-1.514648
H	1.089400	-0.175859	-2.396609
O	0.665608	0.673440	1.284676
H	5.659785	-1.609547	2.186884
O	1.709740	2.192378	-0.649372
H	-0.954501	1.706113	-1.703725
C	0.668216	2.859026	-0.737765
O	0.538763	4.119866	-0.812558
C	-0.727387	2.286635	-0.816395
C	-1.393138	1.997267	0.364090
C	-2.456928	1.044986	0.470363
C	-2.482753	-0.054866	-0.412816
C	-3.465710	-1.006048	-0.282091
C	-4.458600	-0.867833	0.711696
C	-4.426432	0.207733	1.590148
C	-3.414685	1.146432	1.485145
O	-3.584410	-2.137390	-1.022001
O	-5.416639	-1.796846	0.826080

H	-0.750040	3.635855	-0.912474
H	-1.126028	2.558723	1.254746
H	-1.666757	-0.187393	-1.115269
H	-5.189162	0.276646	2.355400
H	-3.373026	1.975499	2.182552
H	-5.263808	-2.471997	0.154062
C	-2.612588	-2.369558	-2.031600
H	-2.663053	-1.592122	-2.800362
H	-1.603908	-2.395117	-1.609316
H	-2.858883	-3.334535	-2.469140

Products4

Al	3.628582	-1.448460	0.640093
O	1.831577	-1.442333	0.217863
O	3.505420	0.186991	-0.208614
O	3.881042	-1.217616	2.320272
H	4.042338	0.976610	-0.150319
H	0.244064	-0.118614	1.367055
H	1.411625	-2.163913	-0.249324
O	4.531773	-2.669755	-0.157962
H	4.639386	-2.789283	-1.096134
Al	1.701844	0.301858	-0.626646
O	0.873802	-0.352556	-2.020955
H	0.818831	0.155553	-2.827041
O	0.446851	0.640923	0.810632
H	4.481196	-1.727325	2.855910
O	1.816637	2.089716	-0.877791
H	-0.787563	1.927477	-1.342827
C	0.920632	3.021786	-0.652580
O	1.161133	4.203157	-0.627949
C	-0.501032	2.488064	-0.447764
C	-0.670080	1.591722	0.778046
C	-1.959546	0.817208	0.772219
C	-2.168541	-0.143717	-0.228600
C	-3.347504	-0.861808	-0.250836
C	-4.341553	-0.626353	0.718214
C	-4.129587	0.321552	1.700831
C	-2.934273	1.038608	1.731116
O	-3.672772	-1.823059	-1.154904
O	-5.493085	-1.329168	0.687376
H	-1.186953	3.327915	-0.340943
H	-0.584963	2.189315	1.688150
H	-1.396902	-0.323830	-0.971554
H	-4.903174	0.486871	2.440517
H	-2.773869	1.776756	2.509606
H	-5.456169	-1.931030	-0.063721
C	-2.754077	-2.090630	-2.199909
H	-2.591732	-1.199859	-2.814641
H	-1.793496	-2.433708	-1.804048
H	-3.203801	-2.876094	-2.803644

TS7

Al	3.691362	-1.438908	0.500655
O	1.866324	-1.462104	0.337253
O	3.402261	0.268332	-0.110556
O	4.283248	-1.461288	2.114409

H	3.923214	1.069246	-0.122754
H	-0.959748	-0.009224	1.336939
H	1.390551	-2.179713	-0.074430
O	4.491600	-2.511478	-0.591533
H	4.382254	-2.505228	-1.537610
Al	1.550800	0.332690	-0.352164
O	0.648211	-0.294856	-1.763443
H	0.758285	0.179460	-2.585554
O	0.370291	0.693650	0.992573
H	4.998856	-2.026718	2.389512
O	1.729242	2.119641	-0.803011
H	-0.889077	1.984225	-1.281186
C	0.838580	3.061063	-0.674712
O	1.056892	4.245405	-0.794389
C	-0.582352	2.548353	-0.395542
C	-0.663671	1.661781	0.848037
C	-1.877761	0.708382	0.905428
C	-2.126789	-0.116686	-0.249794
C	-3.269974	-0.858889	-0.325731
C	-4.238025	-0.766041	0.706004
C	-4.039781	0.064491	1.816628
C	-2.893224	0.810601	1.900583
O	-3.610623	-1.723800	-1.307280
O	-5.337199	-1.496032	0.629357
H	-1.253924	3.399890	-0.281258
H	-0.680786	2.296092	1.741975
H	-1.333144	-0.200186	-0.995466
H	-4.804650	0.093869	2.582147
H	-2.729890	1.465629	2.749461
H	-5.310596	-2.010248	-0.190118
C	-2.707656	-1.861094	-2.403482
H	-2.615449	-0.912805	-2.940253
H	-1.720173	-2.178669	-2.059539
H	-3.141878	-2.616299	-3.053799
Products5			
Al	4.323434	-0.507097	0.060335
O	2.846257	-0.251957	1.154209
O	3.028968	-0.168718	-1.205427
O	5.454062	0.776785	0.160767
H	3.163300	0.441919	-1.930212
H	-3.330968	0.137001	3.051887
H	2.741760	-0.336764	2.098895
O	4.948383	-2.104882	0.124127
H	4.460920	-2.911596	-0.008807
Al	1.508434	0.081224	-0.085301
O	0.426718	-1.208910	-0.525321
H	0.578643	-1.665888	-1.349533
O	0.360661	0.556095	1.504102
H	6.397014	0.657884	0.223851
O	1.162852	1.703785	-0.779729
H	-1.520370	1.103061	-0.440960
C	0.107222	2.468322	-0.717370
O	-0.022858	3.522931	-1.277383
C	-1.053738	1.913107	0.140640

C	-0.607849	1.304232	1.413132
C	-3.485686	0.051735	1.982109
C	-2.959683	-1.049345	1.301116
C	-3.184798	-1.185252	-0.059829
C	-3.922331	-0.206590	-0.751128
C	-4.433344	0.885000	-0.070102
C	-4.218482	1.012163	1.300848
O	-2.771020	-2.212854	-0.841231
O	-4.132780	-0.338443	-2.079308
H	-1.805276	2.680432	0.307517
H	-1.156807	1.542105	2.330952
H	-2.383268	-1.791875	1.837192
H	-5.000941	1.622617	-0.624675
H	-4.634448	1.860983	1.831083
H	-3.707998	-1.154803	-2.364822
C	-2.005104	-3.243970	-0.237723
H	-1.073799	-2.834211	0.162150
H	-2.584688	-3.746806	0.543867
H	-1.784548	-3.954770	-1.032424

Prereaction5

Al	-4.927971	0.747230	0.148908
O	-4.276076	-0.054739	-1.360445
O	-3.583618	-0.234448	0.920955
O	-4.445038	2.395851	0.452020
H	-3.025081	0.199055	1.591538
H	-5.070005	3.116302	0.428701
H	-4.741255	-0.345743	-2.141486
O	-6.592527	0.446746	0.434672
H	-7.033784	-0.394638	0.369093
Al	-2.868936	-1.062041	-0.569558
O	-3.371103	-2.615331	-1.130632
O	-2.354441	1.820041	2.136918
H	-2.869349	-3.402658	-0.937474
H	-2.349574	2.213815	3.008777
O	-1.424324	-0.107937	-1.343467
C	-0.605470	-0.641828	-0.512419
O	-1.119628	-1.455717	0.311341
C	0.810107	-0.305807	-0.548050
C	1.665193	-0.859658	0.322827
C	3.100948	-0.630417	0.425409
C	3.779872	0.273401	-0.411546
C	5.139307	0.455341	-0.276187
C	5.861214	-0.261149	0.698960
C	5.199107	-1.150276	1.524825
C	3.827870	-1.330466	1.386553
O	5.912917	1.296235	-1.016386
C	5.289474	2.070142	-2.022381
O	7.190003	-0.075794	0.823120
H	1.100149	0.408176	-1.308486
H	1.245042	-1.563072	1.038235
H	3.232344	0.829488	-1.161002
H	5.767471	-1.692795	2.270059
H	3.314683	-2.028891	2.038232
H	6.077978	2.663366	-2.481000

H	4.827724	1.429221	-2.780620
H	4.534421	2.736694	-1.592892
H	7.463218	0.575840	0.168152
H	-3.009243	2.313441	1.605908
TS8			
Al	4.820533	0.604028	-0.201031
O	4.248220	0.099908	1.404404
O	3.976545	-0.846944	-0.810176
O	3.599446	1.920066	-0.716169
H	3.549830	-1.025775	-1.648137
H	3.288654	2.474819	0.008018
H	4.698241	-0.165506	2.203050
O	6.383865	1.136469	-0.573759
H	7.225521	0.699192	-0.501629
Al	2.716966	-1.018433	0.700011
O	3.003159	-2.329757	1.779819
O	1.408253	0.932677	-1.430263
H	2.402646	-3.066040	1.855944
H	1.129410	0.547221	-2.264448
O	1.654347	0.465641	0.781671
C	0.938431	0.009937	-0.290163
O	1.349167	-1.255165	-0.550932
C	-0.543509	0.223534	-0.195668
C	-1.418531	-0.755838	-0.409678
C	-2.883545	-0.673141	-0.332622
C	-3.549320	0.471776	0.134198
C	-4.928873	0.507937	0.179969
C	-5.686897	-0.599301	-0.238079
C	-5.038873	-1.731138	-0.692335
C	-3.647831	-1.764695	-0.735565
O	-5.686233	1.557253	0.615848
C	-5.021572	2.716145	1.072269
O	-7.039159	-0.554539	-0.187660
H	-0.835172	1.235093	0.068290
H	-1.008730	-1.728406	-0.672029
H	-2.974821	1.325450	0.469470
H	-5.632392	-2.580876	-1.007427
H	-3.149251	-2.659190	-1.092850
H	-5.800081	3.415298	1.372329
H	-4.382619	2.490958	1.932805
H	-4.417791	3.163679	0.275434
H	-7.291674	0.306551	0.161091
H	2.712989	1.518634	-1.102866
Products6			
Al	-4.802250	0.803774	-0.130718
O	-4.053719	-0.223201	-1.440506
O	-3.962997	-0.428370	0.956729
O	-3.775305	2.207008	-0.007369
H	-3.521427	-0.156246	1.766809
H	-2.125439	1.395413	-0.174715
H	-4.419455	-0.639879	-2.217390
O	-6.493359	1.031494	-0.089310
H	-7.176398	0.377464	0.016846
Al	-2.793520	-1.145501	-0.347484

O	-3.010444	-2.656433	-1.154874
O	-1.354277	0.679332	1.842460
H	-2.422969	-3.387637	-0.981365
H	-0.791275	0.418974	2.575327
O	-1.668842	0.552884	-0.398664
C	-0.894863	-0.028859	0.696783
O	-1.288995	-1.312329	0.676918
C	0.572912	0.207260	0.489131
C	1.444915	-0.795532	0.410107
C	2.898015	-0.699966	0.229972
C	3.544822	0.517054	-0.041114
C	4.916044	0.558620	-0.193780
C	5.682699	-0.615223	-0.080516
C	5.051737	-1.816213	0.179527
C	3.668888	-1.854681	0.330453
O	5.657204	1.671583	-0.462678
C	4.984645	2.904874	-0.609421
O	7.024796	-0.565082	-0.232802
H	0.861867	1.252267	0.430616
H	1.039378	-1.800972	0.492575
H	2.963060	1.424070	-0.141783
H	5.652080	-2.714241	0.259507
H	3.183061	-2.802805	0.533333
H	5.752500	3.646770	-0.820450
H	4.274181	2.869125	-1.442074
H	4.458098	3.177993	0.311314
H	7.268499	0.348886	-0.413052
H	-4.062119	3.114182	0.035052

Preraction6

Al	-4.886029	0.778662	0.165070
O	-4.311920	-0.069622	-1.342388
O	-3.571013	-0.235187	0.942018
O	-4.306043	2.399250	0.480141
H	-2.981609	0.176420	1.599062
H	-4.769976	3.193615	0.230101
H	-4.876459	-0.475740	-1.998627
O	-6.556668	0.441055	0.353344
H	-7.157530	0.816621	0.988379
Al	-2.896451	-1.079057	-0.563163
O	-3.397046	-2.623062	-1.144228
O	-2.212029	1.763767	2.120803
H	-2.911939	-3.419680	-0.947966
H	-2.166028	2.174327	2.983547
O	-1.458006	-0.103717	-1.322186
C	-0.633653	-0.651799	-0.506217
O	-1.139311	-1.485517	0.302212
C	0.780812	-0.308621	-0.541064
C	1.641134	-0.872485	0.317707
C	3.076564	-0.638613	0.419120
C	3.752303	0.265581	-0.419860
C	5.111196	0.452543	-0.285147
C	5.835660	-0.258530	0.691874
C	5.176805	-1.148395	1.519461
C	3.806283	-1.334473	1.381101

O	5.881987	1.293465	-1.028610
C	5.256801	2.056846	-2.041415
O	7.164038	-0.068127	0.815429
H	1.064748	0.420108	-1.289764
H	1.226228	-1.588173	1.023921
H	3.203008	0.816412	-1.171904
H	5.747174	-1.687554	2.265621
H	3.295799	-2.033971	2.033756
H	6.043458	2.649786	-2.503597
H	4.798641	1.408115	-2.795170
H	4.498374	2.723678	-1.618303
H	7.434944	0.581515	0.157563
H	-2.851931	2.278896	1.592933

TS9

Al	-4.814761	0.464567	0.572488
O	-4.321593	0.692631	-1.163003
O	-3.842822	-1.071878	0.405437
O	-3.847364	1.321705	1.756876
H	-3.314818	-1.470634	1.098843
H	-4.076223	2.177788	2.108525
H	-4.909365	0.750896	-1.913108
O	-6.527925	0.472117	0.702764
H	-7.037556	0.522929	1.504733
Al	-2.832107	-0.548692	-1.130216
O	-3.088522	-1.275788	-2.677174
O	-1.236726	0.112495	1.609287
H	-2.495721	-1.947014	-3.003501
H	0.147506	0.478389	1.364574
O	-1.679175	0.811434	-0.609952
C	-0.955447	-0.080091	0.068133
O	-1.265667	-1.315588	-0.301274
C	0.554376	0.286247	0.195455
C	1.466849	-0.723905	0.420135
C	2.893984	-0.663443	0.363527
C	3.579817	0.489951	-0.080388
C	4.952074	0.497961	-0.116329
C	5.680744	-0.646178	0.289284
C	5.014231	-1.782910	0.721461
C	3.630734	-1.789016	0.755547
O	5.744081	1.521845	-0.519923
C	5.124302	2.716866	-0.965016
O	7.016032	-0.627775	0.248258
H	0.845321	1.234337	-0.247248
H	1.031967	-1.681142	0.708477
H	3.018821	1.359149	-0.396464
H	5.594918	-2.645700	1.021679
H	3.105444	-2.675902	1.091290
H	5.931949	3.390576	-1.241773
H	4.491862	2.525316	-1.837321
H	4.529509	3.167837	-0.164644
H	7.300643	0.233774	-0.080138
H	-2.007535	0.710581	1.715310

Products7

Al	3.116613	0.991863	-0.016022

O	2.676010	-0.264908	1.241757
O	2.220715	-0.185291	-1.113300
O	2.155493	2.431346	-0.140083
H	1.599872	0.103588	-1.789676
H	2.415320	3.306800	0.131322
H	3.308858	-0.603811	1.875672
O	4.825282	1.092347	-0.022146
H	5.382833	1.689238	-0.510142
Al	1.828558	-1.527851	0.105031
O	2.838195	-2.859261	0.509760
O	-1.651528	-2.205358	-0.485937
H	2.744624	-3.719944	0.111002
H	-4.803339	-0.166693	2.341114
O	0.049833	-1.369307	0.748030
C	-0.378047	-1.989020	-0.290972
O	0.480140	-2.380925	-1.115272
C	-5.415894	-0.389518	1.473396
C	-4.997338	-0.194460	0.223543
C	-3.690337	0.324658	-0.212741
C	-2.697366	0.726762	0.698099
C	-1.473612	1.196387	0.251597
C	-1.201927	1.265745	-1.129643
C	-2.178727	0.872851	-2.027177
C	-3.409775	0.409449	-1.573834
O	-0.456576	1.605798	1.039847
C	-0.524901	1.340697	2.424980
O	0.002234	1.670841	-1.586960
H	-6.405740	-0.783354	1.669799
H	-5.676004	-0.444371	-0.589076
H	-2.890244	0.679769	1.762153
H	-1.957516	0.924503	-3.086525
H	-4.157865	0.097981	-2.294428
H	0.435578	1.648416	2.833955
H	-1.324799	1.918467	2.901588
H	-0.670700	0.271317	2.603010
H	0.568736	2.052560	-0.880831
H	-2.167676	-1.670173	0.134889
